# Characterization of Normal and Ozone Stressed *Moringa* Mediated Silver (Ag) Nanoparticles (AgNPs) and Plant Crude Extract Against Pathogenic Strains

**DOI:** 10.1002/fsn3.71143

**Published:** 2025-12-17

**Authors:** Misbah Zaid Ali, Zia Ullah, Sabaz Ali Khan, Rafiq Ahmad, Nadia Bibi, Hira Mushtaq, Bushra Rehman, Qamar Sajjad, Awais Raza, Agoura Diantom

**Affiliations:** ^1^ Department of Biotechnology COMSATS University Islamabad, Abbottabad Campus Abbottabad Pakistan; ^2^ Department of Microbiology Abbottabad University of Science & Technology Abbottabad Pakistan; ^3^ Department of Microbiology Shaheed Benazir Bhutto Woman University Peshawar Peshawar Pakistan; ^4^ Center of Biotechnology and Microbiology University of Peshawar Peshawar Pakistan; ^5^ Institute of Pathology and Diagnostic Medicine Khyber Medical University Peshawar Peshawar Pakistan; ^6^ National Institute of Food Science and Technology University of Agriculture Faisalabad Pakistan; ^7^ University Institute of Diet and Nutritional Sciences The University of Lahore Lahore Pakistan; ^8^ Department of Food Science and Technology Universite de Lomé Lomé Togo

**Keywords:** *Moringa oleifera*, nanotechnology, plant extract, repulsion

## Abstract

Nanotechnology is the manipulation of matter at the atomic scale and has evolved as a promising discipline with many applications such as medicine, diagnosis, and sensing devices. Plant extracts consist of many important secondary metabolites that have a tendency to interact with metal oxide to form nanoparticles with peculiar features. Silver nanoparticles (AgNPs) are one of the most vital and fascinating nanomaterials that exhibit antiviral, antibacterial and antifungal characteristics. Therefore, in this study, the medicinal plant that is, *Moringa oleifera* , ozone (stressed and normal) has been used in order to check the inhibition of human pathogenic strains and to synthesize the AgNO_3_ NPs. The confirmation of NPs synthesis was done by UV–vis spectroscopy, Fourier transform infrared spectroscopy, while zeta potential gave the surface charge and stability of NPs. Maximum absorbance peaks were observed in the range of 400 nm. FTIR confirmed that plant secondary metabolites capped and stabilized NPs. The negative zeta potential values of AgNPs showed that nanoparticles have repulsion among themselves, which increases the stability of the formulation. The antibacterial potential of green synthesized nanoparticles and crude extracts of the plants was evaluated against the human pathogenic strains, *S. aureus* and *P. aeruginosa.* It was found that ozone‐stressed plants' NPs inhibit both of the strains while normal plants' NPs inhibit only *P. aeruginosa* . The dual functionality of AgNPs exhibits both antibacterial and potential sensor capabilities. Similarly, the crude extract of the ozone‐stressed plants was able to inhibit both strains while normal plant extract showed inhibition in *S. aureus* at higher concentrations and inhibited *P. aeruginosa* at all concentrations. Our study indicated that *Moringa* plant‐based AgNPs have antibacterial potential as well as that the ozone‐stressed plant‐based NPs were even more effective, which could be beneficial for the synthesis of novel drugs against human pathogens.

## Graphical abstract

Graphical representation of characterization of *Moringa* mediated silver nanoparticles and crude plant extract under normal and ozone stress conditions against pathogenic strains.

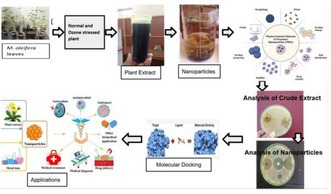

## 1 Introduction

Antibiotic‐resistant infections, such as those caused by MRSA and multidrug‐resistant *E. coli* , are a rising global concern, leading to prolonged illness, higher medical costs, and increased mortality (WHO 2023). In the search for alternative treatments, silver nanoparticles (AgNPs) synthesized using plant extracts like *Moringa oleifera* have gained attention for their potent antimicrobial properties. *Moringa*, rich in phenolics and flavonoids, acts as a natural reducing and stabilizing agent in the green synthesis of AgNPs, which have shown strong antibacterial effects against resistant pathogens (Irfan et al. 2021; Goutam et al. 2020). Recent studies suggest that environmental factors such as ozone stress can alter a plant's phytochemical composition, potentially affecting nanoparticle synthesis and efficacy. Although direct studies on ozone‐stressed *Moringa*‐mediated AgNPs are still limited, preliminary research indicates that ozone may enhance certain bioactive responses in plants, possibly improving the antibacterial strength of the resulting nanoparticles (Rani et al. 2022). These findings highlight the potential of environmentally influenced, plant‐based nanotechnology in developing novel treatments against superbugs.

Antibiotic resistance is becoming an increasingly serious global health concern, making infections harder to treat and leading to more illness, deaths, and healthcare costs. The World Health Organization (WHO) estimates that around 700,000 people die each year due to antibiotic‐resistant infections, and this number could rise dramatically to 10 million annually by 2050 if we don't take action (WHO 2020). Common bacteria like *E. coli* , *Staphylococcus aureus* , and *Klebsiella pneumoniae* are showing resistance to treatments that once worked well, resulting in longer hospital stays and more complicated recoveries. Including these facts helps highlight just how urgent the problem is and why it is so important to explore new treatment options and alternative antimicrobial strategies (WHO 2020).

The control of microbial infection in Egypt, Greece, and China is explained thoroughly. When Sir Alexander Fleming discovered the first antibiotic penicillin the new era of antibiotics started During World War II, penicillin proved to be the best control against bacterial infections. Shortly after this period of time, penicillin resistance became a major threat (Sengupta et al. 2013). One of the most infamous and common bacterial pathogens, *S, aureus* , and yearly causes several hundred thousand to millions of potentially dangerous, invasive infections as well as an undetermined number of mild skin infections (Tong et al. 2015).

*Pseudomonas aeruginosa* is a gram‐negative, aerobic, non‐ spore‐producing rod that may infect both immunocompetent and immunocompromised hosts and cause a range of diseases. *P. aeruginosa* almost always causes the distinctive skin lesion known as ecthyma gangrenosum *P. aeruginosa* ‐induced ecthyma gangrenosum in the groin of a leukemia patient (Alhazmi 2015).

The discovery of antibiotics revolutionized medicine, but the rise of antibiotic resistance has prompted the exploration of nanotechnology in tackling bacterial infections, with strategies like nanoparticle‐enabled antibacterial vaccination, combined or targeted antibiotic delivery, and biofilm disruption. Nanoparticles enhance antibiotic efficacy by improving drug stability, controlled release, and targeted delivery, while also aiding rapid bacterial detection and monitoring, offering promising solutions to overcome the challenges posed by resistant bacteria (Khan and Rasool 2023).

Nanotechnology using nanoscale material has many applications in different fields and is being utilized for clinical applications especially for infectious diseases. Worldwide the infections caused by multi‐drug‐resistant pathogens have a high morbidity and mortality rate. Nanotechnology is emerging as a powerful ally in the fight against antibiotic‐resistant infections, which are becoming increasingly difficult to treat with conventional medicines. Tiny particles, such as silver or zinc oxide nanoparticles, can attack bacteria in several ways at once—by damaging their cell walls, creating harmful oxidative stress, or interfering with essential cellular functions like DNA replication. This multi‐pronged attack makes it much harder for bacteria to develop resistance, compared to traditional antibiotics that usually target just one mechanism (Rai et al. 2012; Durán et al. 2016).

Nanoparticles can also help deliver antibiotics directly to the site of infection, improving the drug's effectiveness while reducing side effects (Hajipour et al. 2012). Importantly, they are able to penetrate protective layers like biofilms, which many bacteria form to shield themselves from treatment (Wang et al. 2017). Even more promising is the use of nanoparticles in combination with existing antibiotics, where they can restore the power of drugs that bacteria have otherwise learned to resist (Lara et al. 2010). These advancements offer a hopeful path forward in developing treatments that can keep up with the rapidly evolving threat of superbugs.

The biological processes for making nanoparticles use a variety of microorganisms, their enzymes, and plant products like isolates and extracts. These approaches have a variety of advantages over traditional physical and chemical processes since they are economical, environmentally friendly, and simple to scale up for mass production. Moreover, green synthesis avoids the use of harmful chemicals, high temperatures, pressures, and energies (Khairulmazmi and Tijjani 2019). *Moringa* contains a rich array of polyphenols, including phenolic acids (such as caffeic, chlorogenic, coumaric, gallic, and ellagic acids), flavonoids like kaempferol glycosides (glucosides, malonyl glucosides, and rutinosides), quercetin, myricetin, epicatechin, rutin, and varying levels of tannins, with the highest concentrations generally found in the leaves (Chiș et al. 2023).

*M. oleifera* has numerous medicinal activities that have been used for centuries in several parts of its native and introduced ranges and its tree has many important roles traditionally in Asian and West African medicine. Numerous tree components have historically been utilized in Indian remedies to treat ascites, rheumatism, poisonous stings, and as cardiac and circulatory stimulants (Schat et al. 2021). Ozone is generally regarded as a pollutant but its effect on the plant showed that it enhances the efficiency of plants by increasing their secondary metabolites; ultimately it enhances the efficacy of nanoparticles. Ozone (O_3_) exposure induces the overproduction of reactive oxygen species (ROS), including hydrogen peroxide (H_2_O_2_), superoxide anions (O_2_^−^), and hydroxyl radicals (•OH).

These ROS function as signaling molecules, triggering the upregulation of both enzymatic and non‐enzymatic antioxidant defense systems in plants. Additionally, O_3_‐induced oxidative stress enhances the biosynthesis of secondary metabolites—such as phenylpropanoids, isoprenoids, and alkaloids—which serve protective roles and constitute the principal bioactive constituents in many medicinal plants. Our biosynthesis method aligns with other green synthesis approaches, such as the use of Equisetum diffusum extract, which also yielded AgNPs with defined plasmonic peaks and antibacterial properties (Fu et al. 2025).

Ozone‐treated NPs showed higher antibacterial activity than normal plant NPs. We also did insilico assessment of virulent proteins of pathogenic strains with *M, oleifera* secondary metabolites, to predict the interaction between the virulent protein and secondary metabolites. *Moringa* and Ag nanoparticles consist of antibacterial activity (Jabbar et al. 2023).

The ozone‐stressed plants of *Moringa* are more efficient than the normal *Moringa* plant and showed high antibacterial activity (Zeng et al. 2020). Therefore, the nanoparticles synthesized from stressed *Moringa* plants can be even more efficient than normal plant nanoparticles (Zhang et al. 2024). So this study has proved that the biological method of nanoparticles synthesis is better than physiochemical methods; it gave the best alternative for antibiotics to inhibit the growth of resistant bacteria (Oyeyinka and Oyeyinka 2018). This research was conducted to check the potential of *Moringa*‐based nanoparticles against two human disease‐causing bacteria. The hypothesis of this research in the form of questions was: (1) How does the *Moringa* plant respond to ozone stress and could nanoparticles synthesized from ozone‐stressed plants give better results against selected pathogens? To achieve this hypothesis, ozone‐stressed plants are treated with ozone stress, nanoparticles are synthesized, characterized and used against pathogenic bacterial strains.

## 2 Material and Methods

### 2.1 Sample Collection

*Moringa* seeds have been collected from Bahria Town Islamabad, sown in COMSATS University Islamabad Abbottabad campus Pakistan. Fresh leaves of *Moringa* were taken from the campus for exact preparation.

### 2.2 Sample Processing

Fresh leaves were collected, first rinsed with tap water, and then the surface was thoroughly cleaned with distilled water while it was running. The *Moringa* samples were initially exposed to ozone in a controlled chamber for approximately 12 days. The ozone concentration was maintained around 100 ppm, which is significantly higher than the natural atmospheric range of 37–45 ppb. The treatment was carried out at a temperature of 25°C, with humidity levels between 80% and 90%, and a daily light cycle of about 10 to 12 h.

### 2.3 Preparation of Plant Extract

**FIGURE 1:**
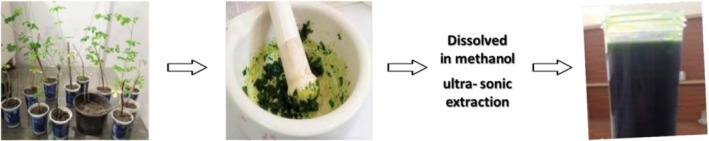
Preparation of plant extract.

For the AgNPs synthesis and for checking the antibacterial activity of NPs and crude extract the aqueous extract of the plant was prepared. In our research 7 g of leaves were taken and cut into small pieces; 50 mL of methanol was added to beakers having leaves. After sonication the extract was centrifuged for 5 min, at 4°C and at 4500 rpm then filtered with filter paper again and again with methanol till the color of the pellet turned brown as shown in Figure 1. The final volume of extract was 20 mL. The extract was kept at 4°C for further use (Bao et al. 2021).

### 2.4 Green Synthesis of Silver Nanoparticles (AgNPs)

To make the 1 M stock solution of AgNOAgNO_3_, 169.9 g of powdered AgNO_3_ was dissolved in 1000 mL distilled water from which a series of solutions were prepared. A 1 M stock AgNO_3_ solution of 10 mL volume was prepared. Different working dilutions of 5 and 10 mM in 50 mL were prepared and mixed with plant crude extract in order to determine the best working dilution for nanoparticles synthesis. Now the flask wrapping was done with aluminum foil (Agarwal and Pal 2024). The extract and AgNO_3_ mixture were incubated at 37°C for 24 h in the dark until a change in the mixture's color from green to brown revealed the presence of silver nanoparticles. The silver nanoparticles (AgNPs) solution was then centrifuged at a speed of 10,000 rpm for 10 min at 25°C. The nanoparticles were cleaned four times with distilled water, and one time with 70% ethanol; the supernatant was discarded, and the pellet of nanoparticles was then dried by freeze‐drying (Agarwal and Pal 2024). The use of *M. oleifera* extract in the green synthesis of AgNPs is consistent with recent approaches utilizing plant‐based systems for eco‐friendly nanoparticle production. For example, AgNPs synthesized using Cotoneaster nummularia under diffused sunlight showed comparable morphological and antimicrobial characteristics (Assad et al. 2025).

### 2.5 Characterization of AgNPs

To truly understand and confirm the successful formation of silver nanoparticles, it's important to characterize them using a few key techniques. UV–Visible spectrophotometry helps us quickly check if the nanoparticles have formed by showing a specific peak (usually around 400–450 nm) that reflects their presence and gives clues about their size. FTIR spectroscopy tells us what kinds of natural compounds, often from plant extracts, are attached to the surface—these not only help in forming the nanoparticles but also keep them stable. Zeta potential analysis measures the surface charge of the particles, which helps us predict how stable they are in suspension; particles with a higher positive or negative charge are less likely to clump together. Altogether, these techniques give us a clear picture of the nanoparticles' structure, stability, and potential for further use.

### 2.6 Ultraviolet–Visible Spectrophotometer

Synthesis of AgNPs was confirmed by spectrophotometer. The 200–700 nm range was used.

### 2.7 Zeta Potential

Zeta potential was used to determine the surface charge potential of the NPs. Surface charge potential is a key factor in determining how stable nanoparticles are in aqueous solutions.

### 2.8 FTIR Analysis of Plant Based AgNPs

FTIR analysis was done to understand the functional group on the surface of plant nanoparticles. An FTIR spectrum was obtained in this region 4000–500 cm^−1^.

### 2.9 Preparation of Sample for Antibacterial Activity of AgNPs

Ag nanoparticles samples were prepared for antibacterial activity against selected pathogenic strains. Three different concentrations of 0.5, 1, and 1.5 mg/mL NPs were dissolved in 1 mL of DMSO; Ciprofloxacin (5 μg) was used as a positive control while DMSO was used as a negative control.

### 2.10 Strains

*S. aureus* (ATCC43300), *P. aeruginosa* (ATCC 15692). For the maintenance of bacterial growth both strains were grown on their respective media. *S. aureus* was grown on Mannitol Salt agar and *P. aeruginosa* was grown on Cetrimide agar plates.

### 2.11 Antibacterial Analysis of AgNPs

Agar well diffusion method was used in order to check the antibacterial activity of AgNPs. The nutrient agar preparation was done according to the company's instructions. After the preparation, the media was autoclaved at 120°C for 20 min; after pouring the media in plates, these plates were kept in an incubator at 37°C for 24 h in order to check the contamination. Then the plates were utilized for antibacterial activity. Twenty‐four‐hour‐old cultures of both strains were used to make the fresh culture of bacteria in nutrient broth.0.5 turbidity McFarland standard which corresponds to10^6^ colony forming units per ml (CFU/mL) was used. The broth media was spread on the plates prepared for antibacterial activity. By using a sterilized gel borer, 6‐mm diameter wells have been created on agar plates. In separate wells different dilutions of extract were added (0.5, 1, 1.5 mg/mL). DMSO was used as a negative control while ciprofloxacin disc (antibiotic) was used as a positive control. The wells were loaded with 100 μL of each sample. The plates were kept for 24 h in an incubator. The zone of inhibition was measured after 24 h of incubation at 37°C. Each organism's plates were kept in triplicate (Asif et al. 2022).

### 2.12 Preparation of Sample for Antibacterial Activity of Plant Crude Extract

The samples of crude extract were prepared against the pathogenic strains. Different concentrations, for example, 0.5, 1, 1.5, 5, 10, 30, 50, 100, and 300 mg of plant crude extract were dissolved in DMSO (dimethyl sulfoxide) to check the antibacterial activity of the plant. Ciprofloxacin (5 microgram) was used as a positive control while DMSO was used as a negative control. Using the agar well diffusion method, the crude extracts' antimicrobial activity was assessed. The nutrient agar preparation was done according to the company's instructions. After the preparation the media was autoclaved at 120°C for 20 min; after pouring the media into plates, these plates were kept in an incubator at 37°C for 24 h in order to check for contamination. Then the plates were utilized for antibacterial activity. Twenty‐four‐hour‐old cultures of both strains were used to make the fresh culture of bacteria in nutrient broth. The 0.5 turbidity McFarland standard which corresponds to 10^6^ colony forming units per ml (CFU/mL) was used. The broth media was spread on the plates prepared for antibacterial activity. By using a sterilized gel borer, 6‐mm diameter wells were created on agar plates. In separate wells different dilutions of extract were added (0.5, 1, 1.5, 5, 30, 50, 100 and 300 mg/mL). DMSO was used as a negative control while the ciprofloxacin disc (antibiotic) was used as a positive control. The wells were loaded with 100 μL of each sample. The plates were kept for 24 h in an incubator; each organism's plates were kept in triplicate (Al Akeel et al. 2014).

### 2.13 Estimation of Total Phenolic Contents (TPC)

The determination of total phenolics was done by spectrophotometer‐based Folin–Ciocalteu (FC) colorimetric in vitro assay as described earlier (Babbar et al. 2011) with some changes. To an aliquot of sample (500 μL), the 10‐fold diluted FC reagent (1 mL) was added and kept for 6 min; after that 2 mL of 20% sodium carbonate (Na_2_CO_3_) was mixed, and the mixture was allowed to keep for the next 1 h at 30°C under dark conditions. Next the absorbance was measured against a blank at 760 nm wavelength by UV–visible spectrophotometer (UV‐1100). The experiment was carried out in triplicates. The measured TPC was expressed as mg Gallic acid equivalents in 100 g sample (mg GAE/100 g, DW).

### 2.14 Estimation of Total Flavonoid Contents (TFC)

TFC was determined using an aluminum chloride colorimetric assay as discussed earlier (Wibowo and Surono 2024). First of all, 250 μL of each sample in triplicate was added to falcons, along with 1250 μL of distilled water followed by the addition of 75 μL of 5% sodium nitrite (NaNO_2_) and held for 6 min. Next 150 μL of 10% aluminum chloride (AlCl_3_) was added to the solution and kept for 5 min; after that 500 μL of 1 M sodium hydroxide (NaOH) was added to stop the reaction. For the dilution of the solution 275 μL of distilled water was added and immediately measured for absorbance at 510 nm against blank by UV/vis spectrophotometer. The final results were expressed as mg quercetin dihydrate equivalents (QDE)/100 g weight (DW) (Wibowo and Surono 2024).

### 2.15 In Silico Assessment Using Molecular Docking

I used molecular docking to see how plant secondary metabolites might interact with the pathogenic proteins. This helped me understand which compounds could potentially block or weaken the pathogens, giving insight into their possible antimicrobial effects.

The Protein Data Bank (PDB; www.rcsb.org) was accessed in order to evaluate medicinal plants against targeted human pathogens utilizing a molecular docking approach and the Molecular Operating Environment (MOE 2009.10) tool. Last but not least, interactions between reported phenolics and flavonoids of the chosen plants and virulent targets of the diseases under study were characterized and examined using the Molecular Operating Environment (MOE) software. (www.chemcomp.com/Products.htm).

### 2.16 Ligands Optimization and Database Construction

A database that contains hundreds of compounds along with information on their characteristics and bioactivities, PubChem (pubchem.ncbi.nlm.nih.gov), was used to retrieve the 3D structures of FDA‐approved phenolics and flavonoids that are frequently found in the plants under study. The ligands were redrawn in MOE software, hydrogen was added, and force field MMFF94x and a 0.0001 gradient were used to minimize energy. The MOEW database was then updated with optimized ligands (Agarwal and Pal 2024).

### 2.17 Proteins Preparation for Docking

Targeted pathogenic proteins' 3D chemical structures were acquired from Alpha Fold. Then, using the default settings for MOE algorithms, proteins were optimized by eliminating water molecules, protonating 3D (electrostatic: GB/VI; dielectric: 1; van der Waals: 800R3), and minimizing energy (forcefield: MMFF94x; gradient: 0.05). The active sites of proteins were specified for docking using the MOE active site finder and sequence editor (Kar et al. 2024).

### 2.18 Molecular Docking

Utilizing the MOE docking technique, docking simulations were carried out utilizing a number of parameters, including placement: Triangle Matcher, Refinement: Forcefield, Rescoring function 1: London dG, Retain: 10, Rescoring function 2: London dG, and Retain: 10. Analysis of the docking findings was conducted using minimum S‐scores and RMSD values. Additionally, the optimum ligand conformations were chosen based on the binding interactions (Kar et al. 2024).

### 2.19 Statistical Analysis

All the readings were carried out in triplicate and values were expressed as mean ± standard deviation (SD). Data were analyzed using one‐way ANOVA followed by Tukey's post hoc test. Differences were considered statistically significant at *p* < 0.05.

## 3 Results

### 3.1 Physical Observation of Silver Nanoparticles (AgNPs)

**FIGURE 2:**
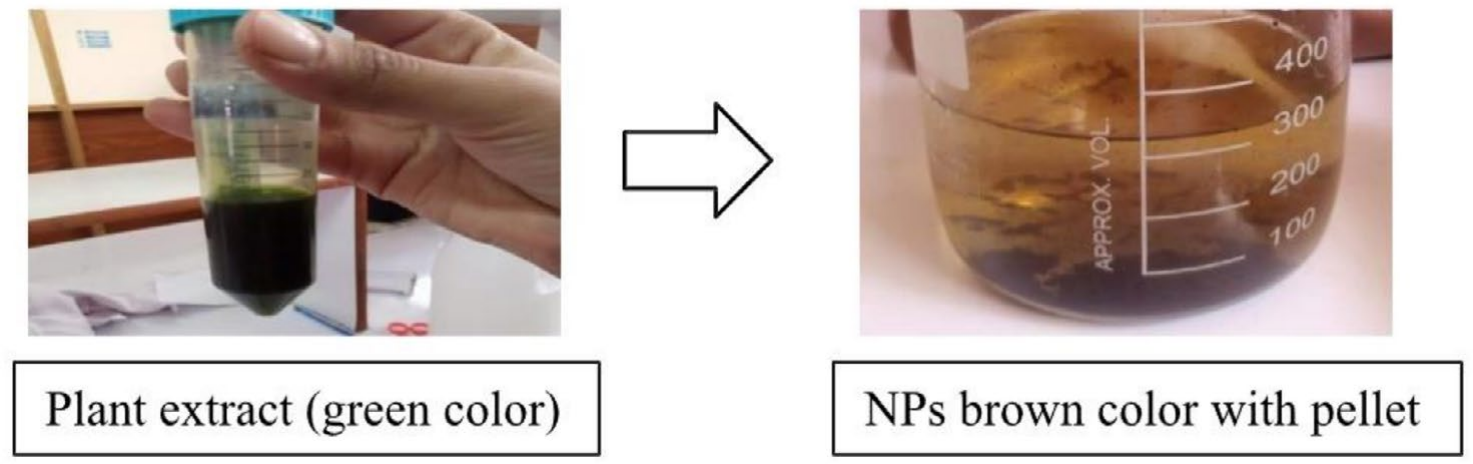
Biosynthesized AgNO_3_ NPs using *M. oleifera* plant extract.

When the plant extract was mixed with Ag solution for the synthesis of AgNPs the overall mixture was green, After 24 h the solution changed its color from greenish to brownish and the formation of a pellet occurred at the bottom of the reagent bottle. This change in color from deep green to brownish as shown in Figure 2 is a clear indication that the reaction had completed. The successful biosynthesis of Ag nanoparticles, as confirmed by characteristic surface plasmon resonance peaks, is consistent with nanostructure synthesis methods previously explored for Ag NP‐loaded composites (Cao et al. 2025), indicating stable formation and effective dispersion.

### 3.2 Analysis of AgNPs Synthesis Through UV–Visible Spectrophotometer

**FIGURE 3:**
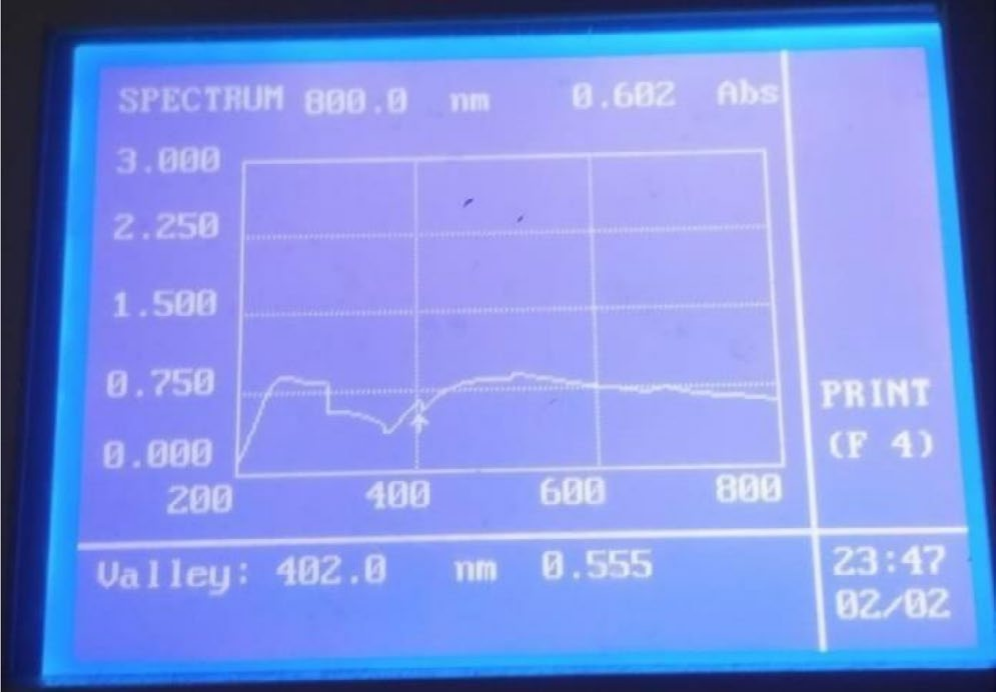
UV–visible spectrum of aqueous solution AgNPs synthesized from *M. oleifera* plant.

UV–Visible spectrum of AgNPs was obtained through UV–Visible spectrophotometer as shown in Figure 3. The peak of synthesized NPs lies in the range of 400 nm, which confirmed the formation of silver nanoparticles. This peak is typical for their surface plasmon resonance.

### 3.3 Analysis of AgNPs Synthesis Through Zeta Potential

**FIGURE 4:**
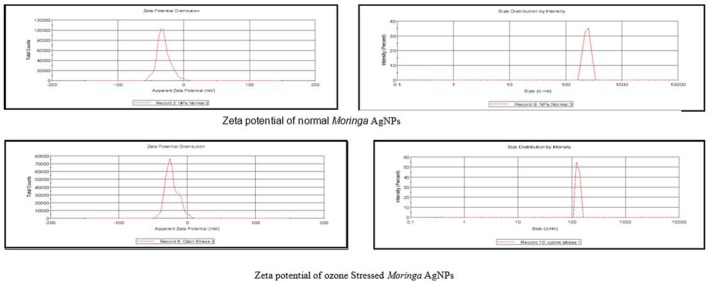
Zeta potential of normal and ozone‐stressed *Moringa* AgNPs.

The AgNPs' surface potential and particle size have been determined using zeta potential as shown in Figure 4. Normal *Moringa* AgNPs were discovered to have particle diameters of 241 ± 2.04 nm. AgNPs' zeta potential values were measured at −30 mV. It was claimed that the surfaces of the generated nanoparticles were negatively charged. Ozone‐stressed *Moringa* AgNPs were found to be 131 ± 9 nm in size. AgNPs from an ozone‐stressed *Moringa* plant had a zeta potential value of −23 mV.

The results in Figure 4 show both the size and surface charge (zeta potential) of the silver nanoparticles (AgNPs) we made from *Moringa* extracts. In the case of normal *Moringa*, the particles were larger—about 241 ± 2.04 nm—with a zeta potential of −30 mV, which tells us they were quite stable and had negatively charged surfaces. This negative charge likely comes from plant compounds that coat and stabilize the nanoparticles. On the other hand, the AgNPs made from ozone‐treated *Moringa* were much smaller, around 131 ± 9 nm, and had a slightly lower zeta potential of −23 mV. This suggests that ozone exposure may have helped break down the plant compounds more effectively, leading to smaller particles. Although the surface charge was a bit less negative, the particles were still stable. Overall, the ozone treatment seems to have a clear impact—producing smaller, stable AgNPs that may have stronger biological effects.

### 3.4 Analysis of AgNPs Synthesis Through FTIR

**FIGURE 5:**
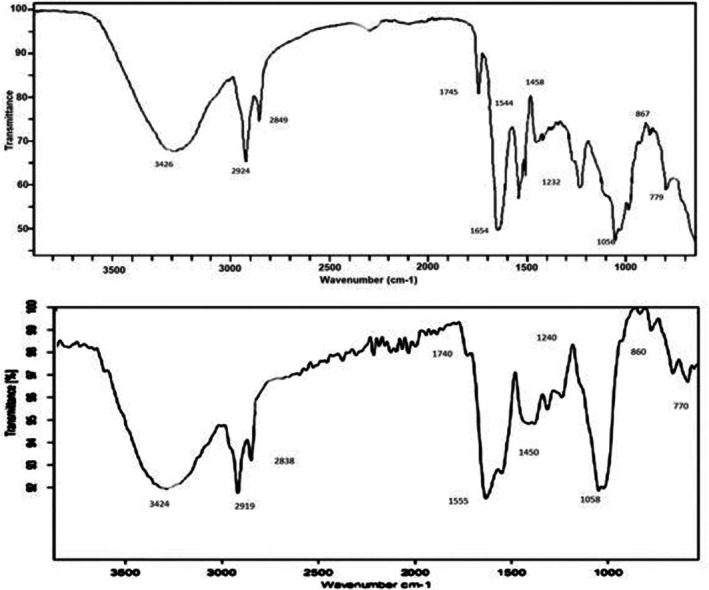
FTIR spectra of normal and ozone‐treated Ag NPs.

Fourier Transform Infrared (FTIR) spectroscopy is a commonly used method for identifying the functional groups present in a substance by analyzing how it absorbs infrared light. Each peak in an FTIR spectrum corresponds to the vibration of specific chemical bonds, such as stretching or bending. For instance, a broad peak between 3200 and 3600 cm^−1^ often indicates the presence of O–H bonds, commonly found in alcohols or carboxylic acids. A sharp peak near 1700 cm^−1^ usually points to C=O bonds, which are typical in ketones, aldehydes, or acids. Peaks around 2900 cm^−1^ are generally linked to C–H bonds in alkanes, while those between 1500 and 1600 cm^−1^ may reflect vibrations from aromatic rings. Overall, the FTIR spectrum serves as a kind of molecular fingerprint, allowing researchers to detect and confirm functional groups and track chemical changes in a sample. (Smith 2011; Stuart 2004). The FTIR spectrum was done to confirm the functional group of the nanoparticles. These functional groups facilitate the reduction of silver to produce nanoparticles with certain structures and functions. Significant peaks observed on FTIR spectra were then compared with the standard FTIR table to understand the functional groups present in plant metabolites that served as capping agents during the synthesis of AgNPs. The FTIR spectrum of normal plant‐based NPs showed strong peaks at 770, 860, 1058, 1240, 1450, 1555, 1740, 2838, 2919 and 3424 cm as shown in Figure 5. The FTIR spectrum of ozone‐treated NPs showed a strong absorption band at 779, 867,1056, 1232,1458, 1544, 1654, 1745, 2849, 2924 and 3426 cm as shown in Figure 5.

FTIR analysis revealed that both normal and ozone‐treated *Moringa* AgNPs contained similar functional groups, such as O–H, C–H, and C=O, which are known to aid in the reduction and stabilization of nanoparticles. However, the ozone‐treated sample showed some shifted and additional peaks (e.g., at 1654 cm^−1^), indicating that ozone exposure may have slightly altered the plant compounds, making them more reactive. This modification could be the reason why the ozone‐treated AgNPs were smaller in size yet remained stable, suggesting improved formation and potentially enhanced biological activity.

### 3.5 Antibacterial Activity of AgNPs of *M. oleifera*

To assess the antibacterial potential of silver nanoparticles (AgNPs), we synthesized them using two AgNO_3_ concentrations: 5 and 10 mM. This was a preliminary step to identify the most effective working concentration. When tested against two bacterial strains, the 5 mM AgNPs showed no zone of inhibition against one strain, indicating limited activity. In contrast, the 10 mM AgNPs demonstrated clear antibacterial effects against both strains. Based on these results, we selected 10 mM as the working concentration for further experiments.

**FIGURE 6:**
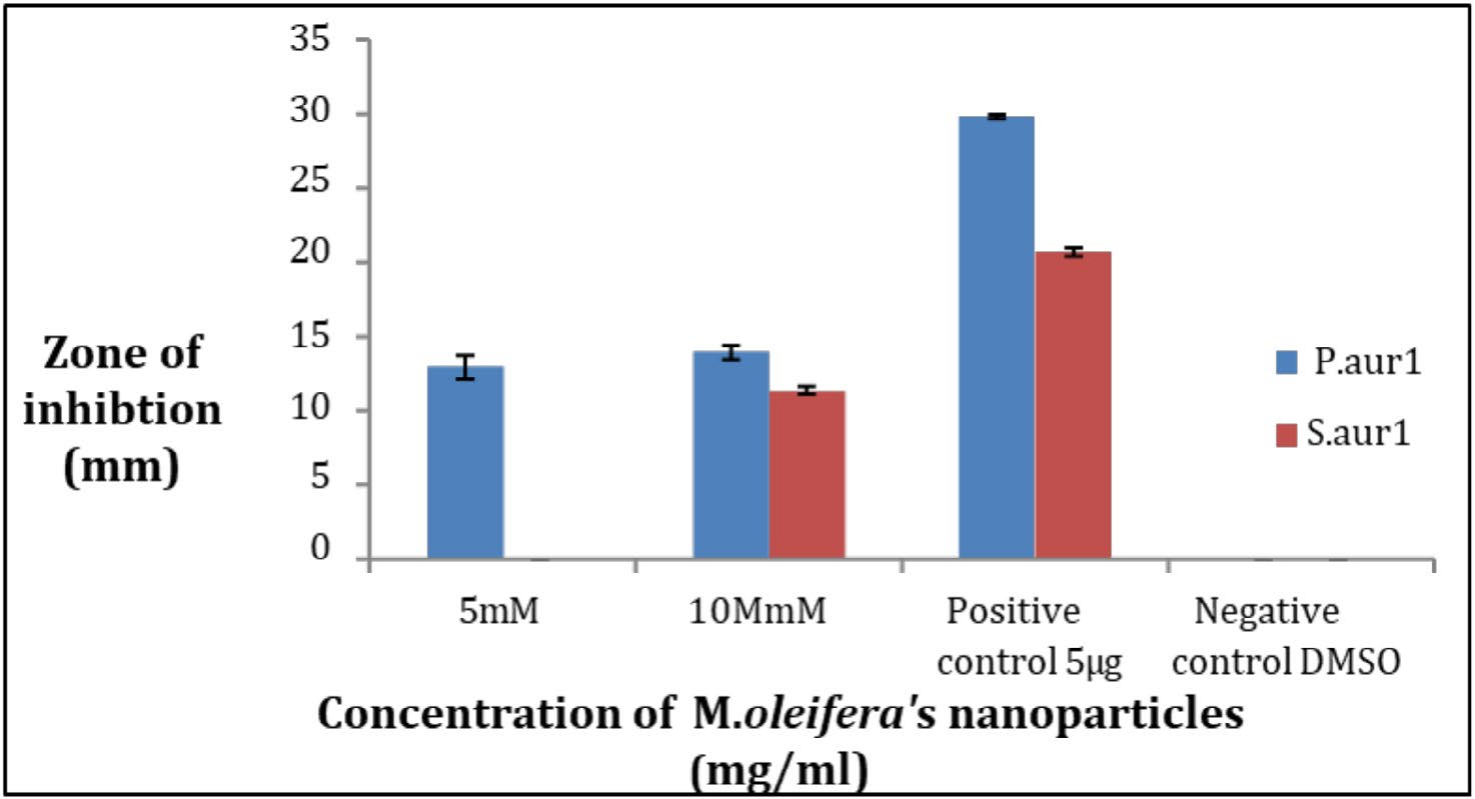
Comparison of antibacterial activity between *P. aeruginosa* and *S. aureus* at 5 and 10 mM concentrations.

Antibacterial activity is found in AgNPs which are synthesized from the extract of *M. oleifera* against the selected pathogenic strains. The results show that a nanoparticle of silver shows activity at two different concentrations, namely 5 and 10 mM, when compared to the common antibiotic ciprofloxacin as shown in Figure 6. The maximum zone of inhibition for *P. aeruginosa* was 14 ± 0.8 mm wide at 10 mM while the maximum zone for *S. aureus* was 11.3 ± 0.4 mm wide at 10 mM, while at 5 mM, *S. aureus* gave no results but *P. aeruginosa* showed a zone of inhibition. Each result was performed in triplicates and their corresponding mean value was taken. The negative control did not give any activity. Therefore, it can be assumed that pathogenic bacterial strains can be killed by AgNPs synthesized from the extract of *M. oleifera* .

To ensure the reliability of our results, all experiments were carried out in triplicate. For each set of data, we calculated the mean to represent the central trend, and then used the standard deviation (±SD) to show how much variation existed between the three replicates. This helped us assess how consistent the results were within each group. In some cases, we also calculated the standard error (SE) to better reflect the precision of the mean. The final results are presented as mean ± SD, and we used one‐way ANOVA followed by Tukey and LSD post hoc tests to compare differences between groups, considering results statistically significant at *p* ≤ 0.05.

### 3.6 Preliminary Screening of AgNPs Concentrations

To identify the most effective working concentration of silver nanoparticles (AgNPs), we initially tested 0.5, 1.0, and 1.5 mg/mL against two bacterial strains. This preliminary screening was intended to determine the lowest concentration showing clear antibacterial activity. The results helped guide the selection of a suitable working range for further analysis.

### 3.7 Comparison of Antibacterial Activity of Ozone‐Stressed and Untreated *M. oleifera* 's Extract AgNPs for *S. aureus* at the Concentrations 0.5, 1, and 1.5 mg/mL

**FIGURE 7:**
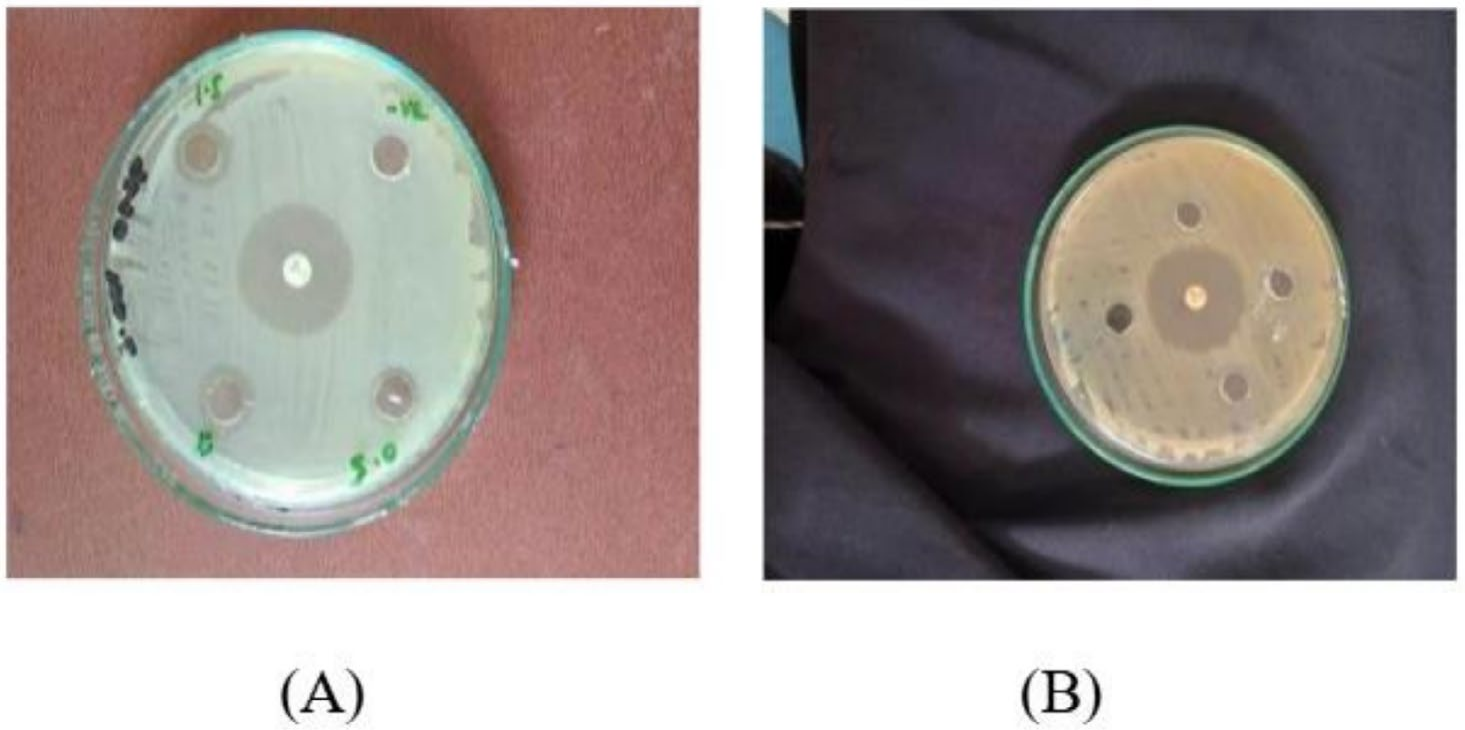
Zone of inhibition at 0.5, 1, and 1.5 mg/mL of *M. oleifera* NPs for pathogenic strain, *S. aureus*. (A) Treated; (B) untreated.

**FIGURE 8:**
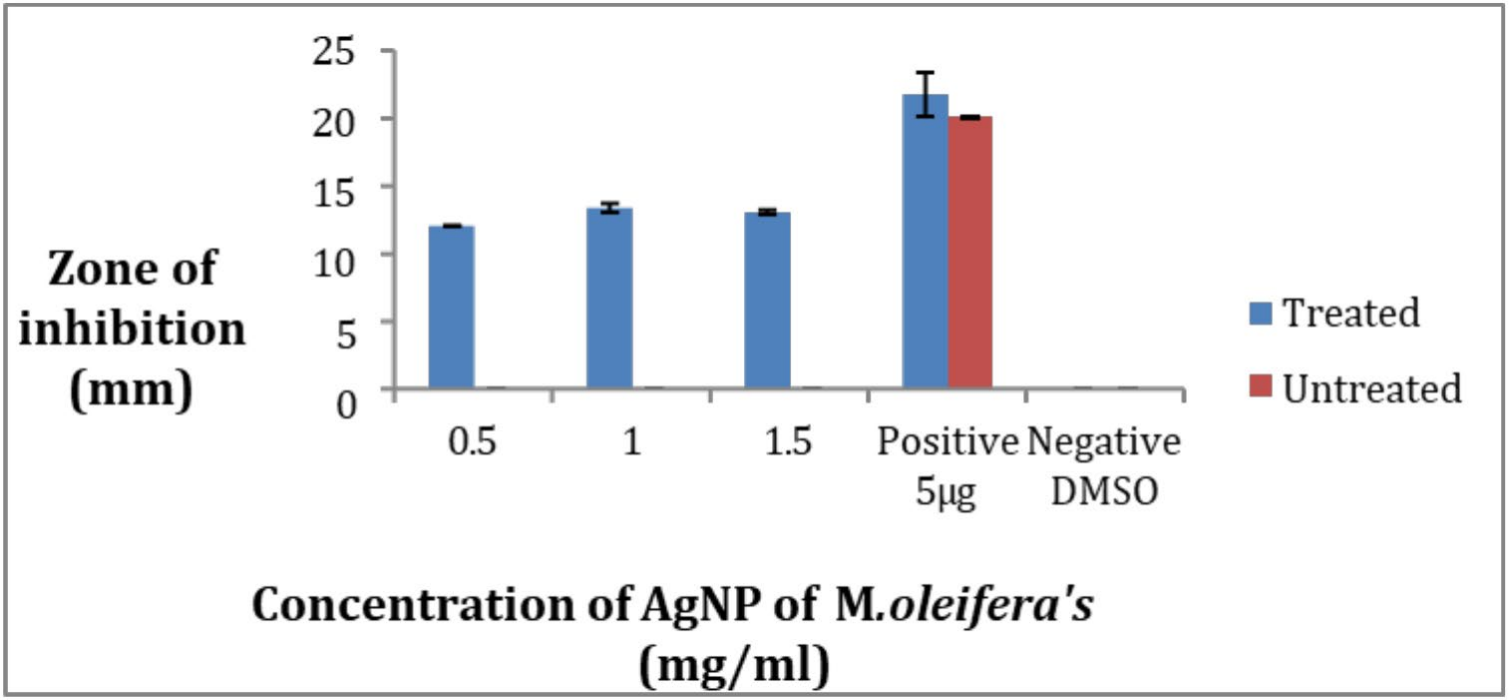
Zone of inhibition of *S. aureus* strain by AgNP of *Moringa oleifera*.

The results show that AgNPs of *M. oleifera* have activity against pathogenic strains. By the comparison of AgNPs synthesized from the ozone‐stressed *M. oleifera* leaves and untreated *M. oleifera* leaves it confirms that ozone‐stressed leaves extract NPs showed more antibacterial activity than untreated leaves extract NPs, as in Figure 7 in comparison with the standard antibiotic ciprofloxacin. When comparing the concentrations 0.5, 1, and 1.5 mg/mL for both treated and untreated leaves extract NPs the maximum zone of inhibition was 13.3 ± 0.5 mm at the concentration 1 mg/mL of treated while the minimum zone was 12 ± 0.05 mm at 0.5 mg/mL of treated, but untreated did not give any activity at all at the three concentrations (Figure 8). So the ozone‐stressed *M. oleifera* leaves NPs are found to be more effective against *S. aureus* than untreated *M. oleifera* leaves NPs. Negative control did not give any activity.

### 3.8 Comparison of Antibacterial Activity of Ozone‐Stressed and Untreated *M. oleifera* 's Extract AgNP for *P. aeruginosa* at the Concentration 0.5, 1, and 1.5 mg/mL

**FIGURE 9:**
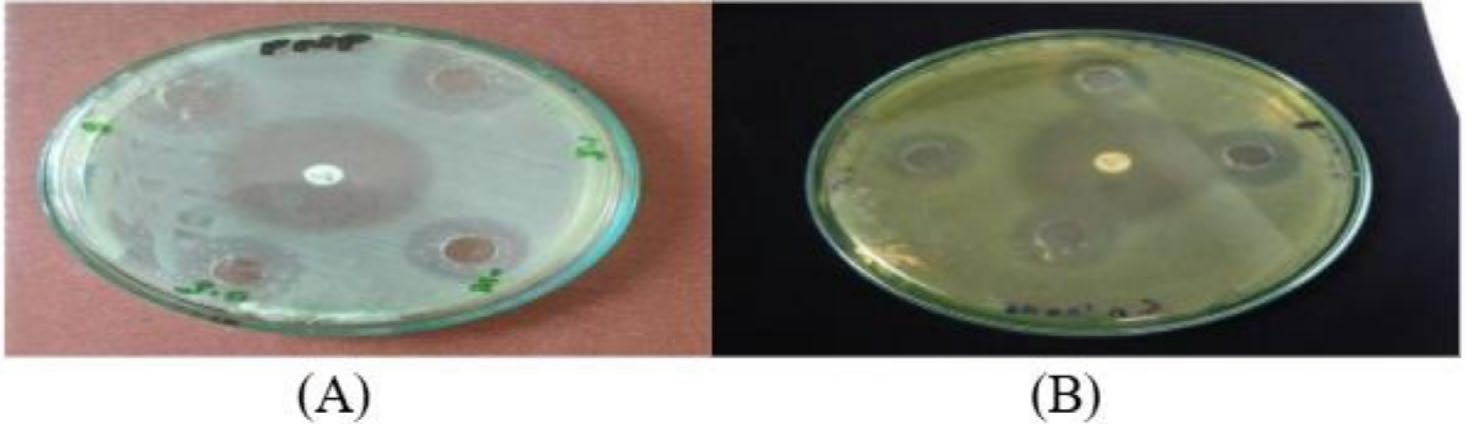
Zone of inhibition at 0.5, 1, and 1.5 mg/mL of *M. oleifera* NPs for pathogenic strain *P. aeruginosa* (A) treated, (B) untreated.

**FIGURE 10:**
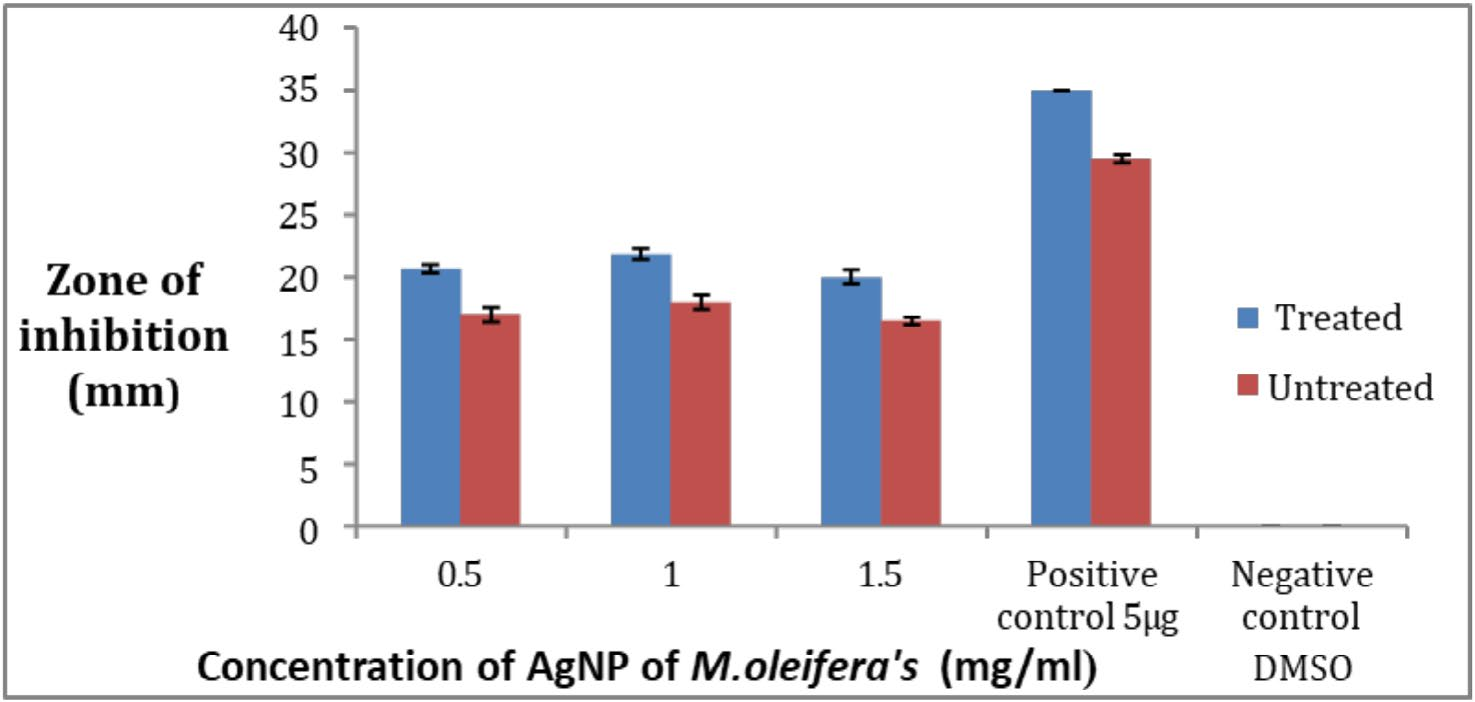
Zone of inhibition of *P. aeruginosa* strain by nanoparticles of *Moringa oleifera*.

The results show that AgNPs of *M. oleifera* have activity against pathogenic strains. By the comparison of AgNPs synthesized from the ozone‐stressed *M. oleifera* leaves and untreated *M. oleifera* leaves it is proven that ozone‐stressed leaves extract NPs showed more antibacterial activity than untreated leaves extract NPs (Figure 9) in comparison with the standard antibiotic ciprofloxacin. When comparing the concentrations 0.5, 1, and 1.5 mg/mL for both treated and untreated leaves extract NPs the maximum zone of inhibition was 21.833 ± 0.7 mm at the concentration of 1 mg/mL of treated while the minimum zone was 20 ± 1 mm at 1.5 mg/mL of treated; the maximum zone of inhibition was 18 ± 1 mm at 1 mg/mL and the minimum zone was 16.5 ± 0.5 mm at 1.5 mg/mL. Thus, the ozone‐stressed *M. oleifera* leaves NPs are found to be more effective against *P. aeruginosa* than untreated *M. oleifera* leaves NPs (Figure 10). The negative control did not give any activity.

A one‐way ANOVA revealed a statistically significant difference in antimicrobial activity across all tested groups. For *Staphylococcus aureus* , the treated nanoparticles showed notably stronger effects, with the 1.0 mg/mL ozone‐treated group producing the highest inhibition zone of 13.3 ± 0.50 mm (95% CI: ±0.566 mm), followed by 0.5 mg/mL at 12.0 ± 0.05 mm (95% CI: ±0.057 mm), while untreated nanoparticles showed no activity at any concentration tested (*F* = 2548.40, *p* < 0.0001). Similarly, against *Pseudomonas aeruginosa* , the results were consistent—ANOVA confirmed a significant difference (*F* = 32.13, *p* < 0.000001), and Tukey's post hoc test indicated that all concentrations of ozone‐treated nanoparticles had significantly higher inhibition zones than their untreated counterparts (*p* < 0.05). The most potent effect was again observed at 1.0 mg/mL, with a zone of 22.08 ± 0.71 mm (95% CI: ±0.80 mm), while the untreated group at the same concentration showed a much smaller zone of 16.87 ± 1.19 mm (95% CI: ±1.35 mm). Altogether, these findings provide strong statistical evidence supporting the superior antimicrobial efficacy of ozone‐stressed nanoparticles against both bacterial strains.

### 3.9 Rationale Behind Selection of Multiple Crude Extract Concentrations

A wide range of crude extract concentrations (0.5–300 mg/mL) was used to evaluate and compare the antibacterial activity of ozone‐treated and untreated *Moringa* leaves. This approach allowed us to observe minimum inhibitory effects and dose‐dependent responses. Overlapping concentrations were intentionally included based on the expected higher potency of the ozone‐treated samples.

### 3.10 Comparison of Antibacterial Activity of Ozone‐Stressed and Untreated *M. oleifera* 's Crude Extract for *S. aureus* at the Concentration 0.5, 1, and 1.5 mg/mL

**FIGURE 11:**
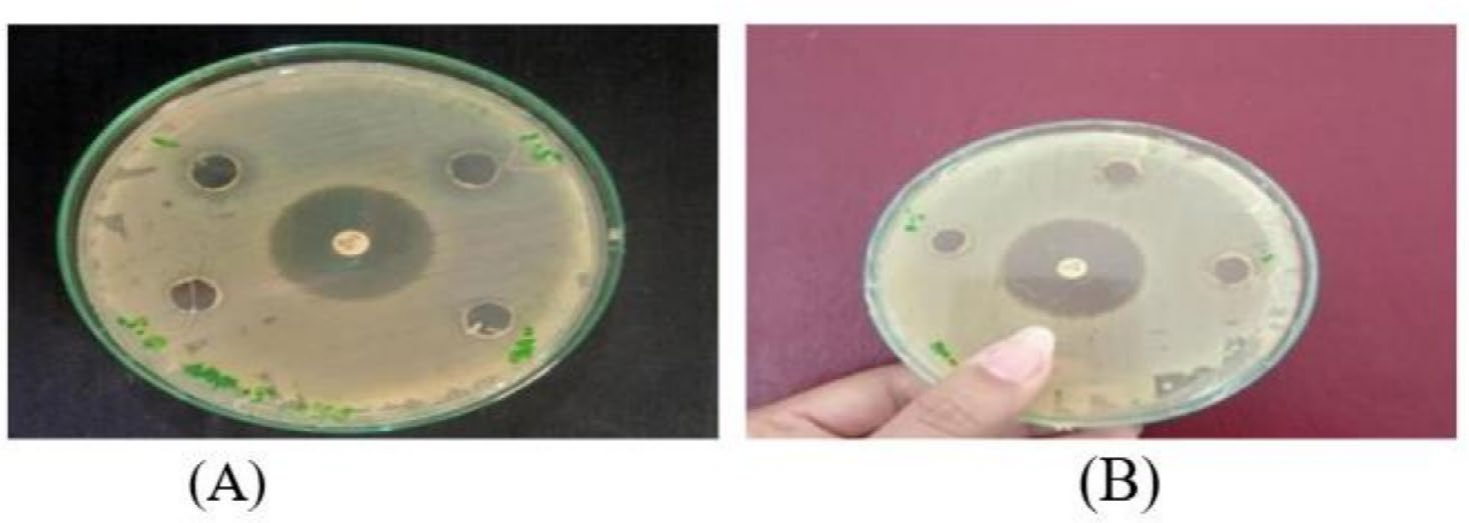
Zone of inhibition at 0.5, 1, and 1.5 mg/mL of *M. oleifera* crude extract for pathogenic strain *S. aureus* (A): treated, (B): untreated.

**FIGURE 12:**
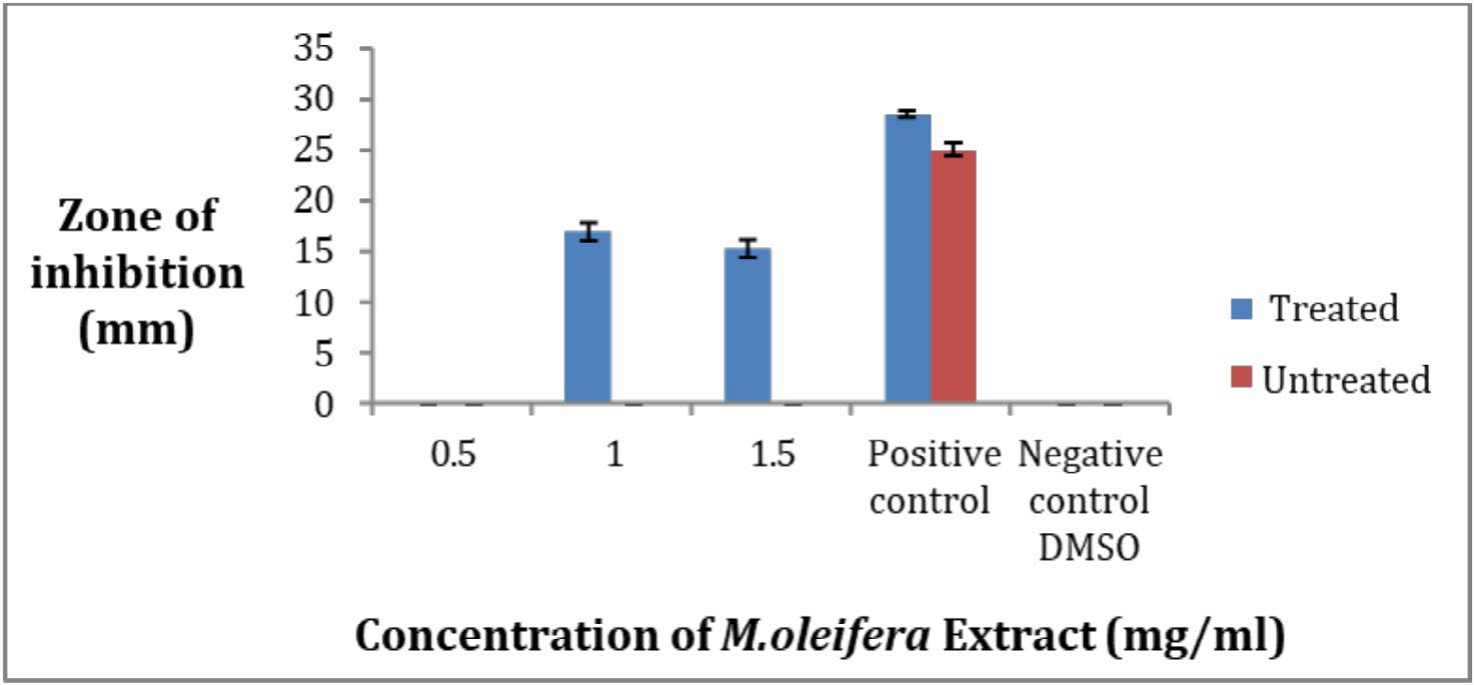
Zone of inhibition of *S. aureus* strain by crude extract of *Moringa oleifera*.

The results show that the crude methanolic extract of *M. oleifera* leaves has antibacterial activity. When the ozone‐treated and untreated leaves extract were compared at concentrations 0.5, 1, and 1.5 mg/mL in comparison with the antibiotic ciprofloxacin, the treated extract showed activity (Figure 11). The maximum zone of inhibition was 16.6 ± 1.5 mm at 1 mg/mL while the minimum was 0 mm at 0.5 mg/mL for the ozone‐treated leaves extract while no activity was found for the untreated at all three concentrations (Figure 12). Each result was performed in triplicates and the mean was taken. So it can be assumed that the illness‐causing strain *S. aureus* can be killed by the crude extract of *M. oleifera*. Negative values did not give activity.

### 3.11 Comparison of Antibacterial Activity of Ozone‐Stressed and Untreated *M. oleifera* 's Crude Extract for *P. aeruginosa* at the Concentration 0.5, 1, and 1.5 mg/mL

The ozone‐treated and untreated leaves' crude extract showed antibacterial activity at concentrations of 0.5, 1, and 1.5 mg/mL in the comparison of the standard antibiotic ciprofloxacin.

**FIGURE 13:**
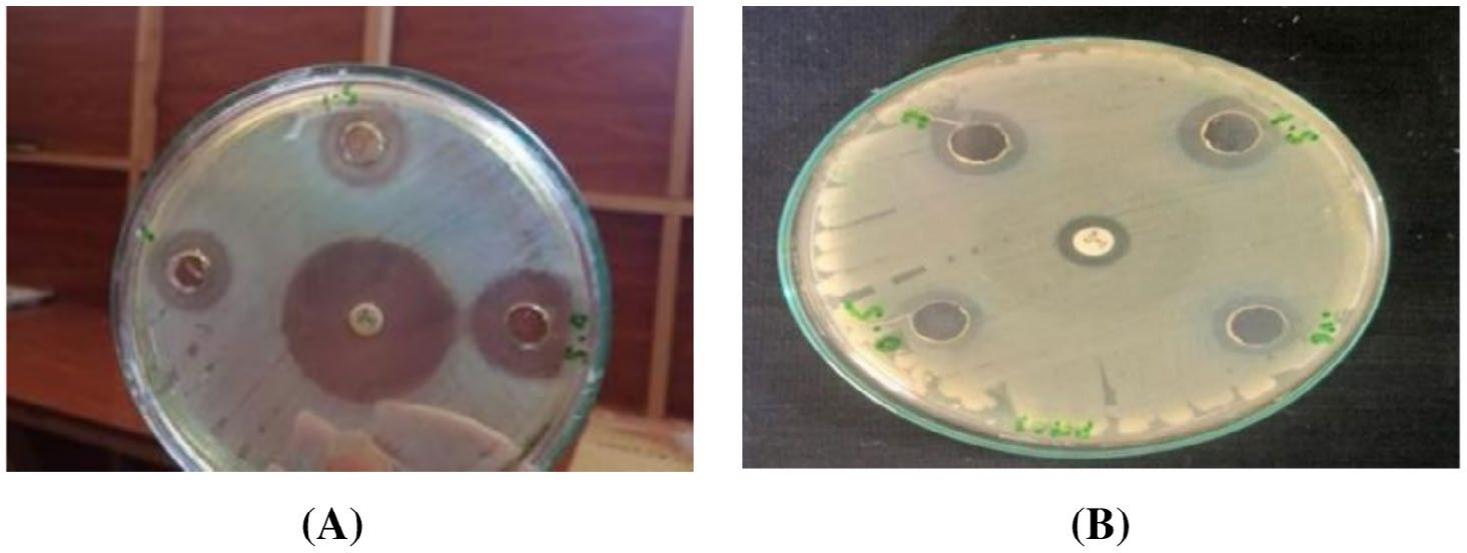
Zone of inhibition at 0.5, 1, and 1.5 mg/mL of *M. oleifera* crude extract for pathogenic strain *P. aeruginosa* (A) treated, (B) untreated.

**FIGURE 14:**
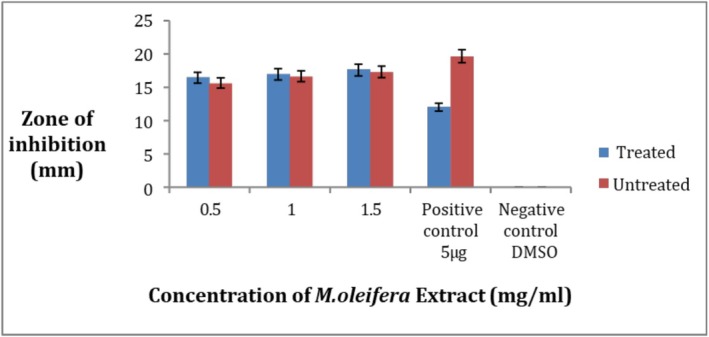
Zone of inhibition of *P. aeruginosa* strain by crude extract of *Moringa oleifera*.

The maximum zone of inhibition in the case of ozone‐treated leaves extract was 17.66 ± 1.5 mm at 1.5 mg/mL and the minimum zone was 16.5 ± 0.5 at a concentration of 0.5 mg/mL (Figure 13). In the case of untreated plant extract the maximum zone was 17.33 ± 2 at 1.5 mg/mL while the minimum zone of inhibition was 15.66 ± 1.1 at 0.5 mg/mL as shown in Figure 14. As a whole the ozone‐treated leaves extract is more effective than the untreated leaves extract. All the values were taken in triplicates. The negative control did not give any value.

### 3.12 Comparison of Antibacterial Activity of Ozone‐Stressed and Untreated *M. oleifera* 's Crude Extract for *S. aureus* at the Concentrations 5, 10, and 30 mg/mL

**FIGURE 15:**
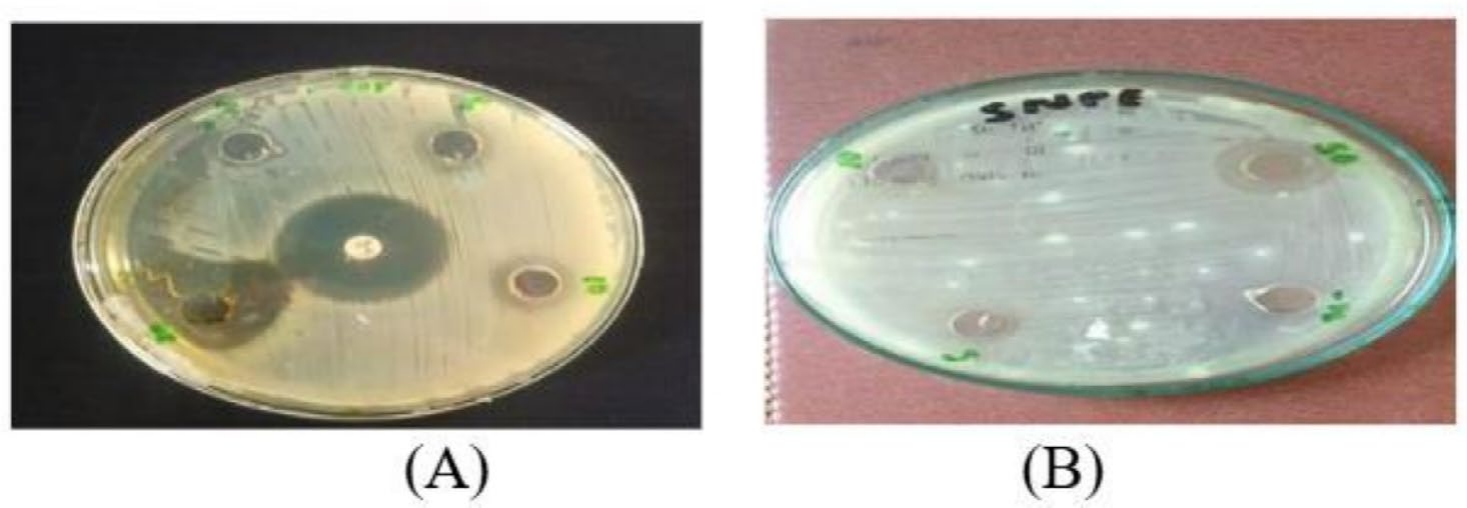
Zone of inhibition at 5, 10, and 30 mg/mL of *M. oleifera* crude extract for pathogenic strain, *S. aureus* (A) treated, (B) untreated.

**FIGURE 16:**
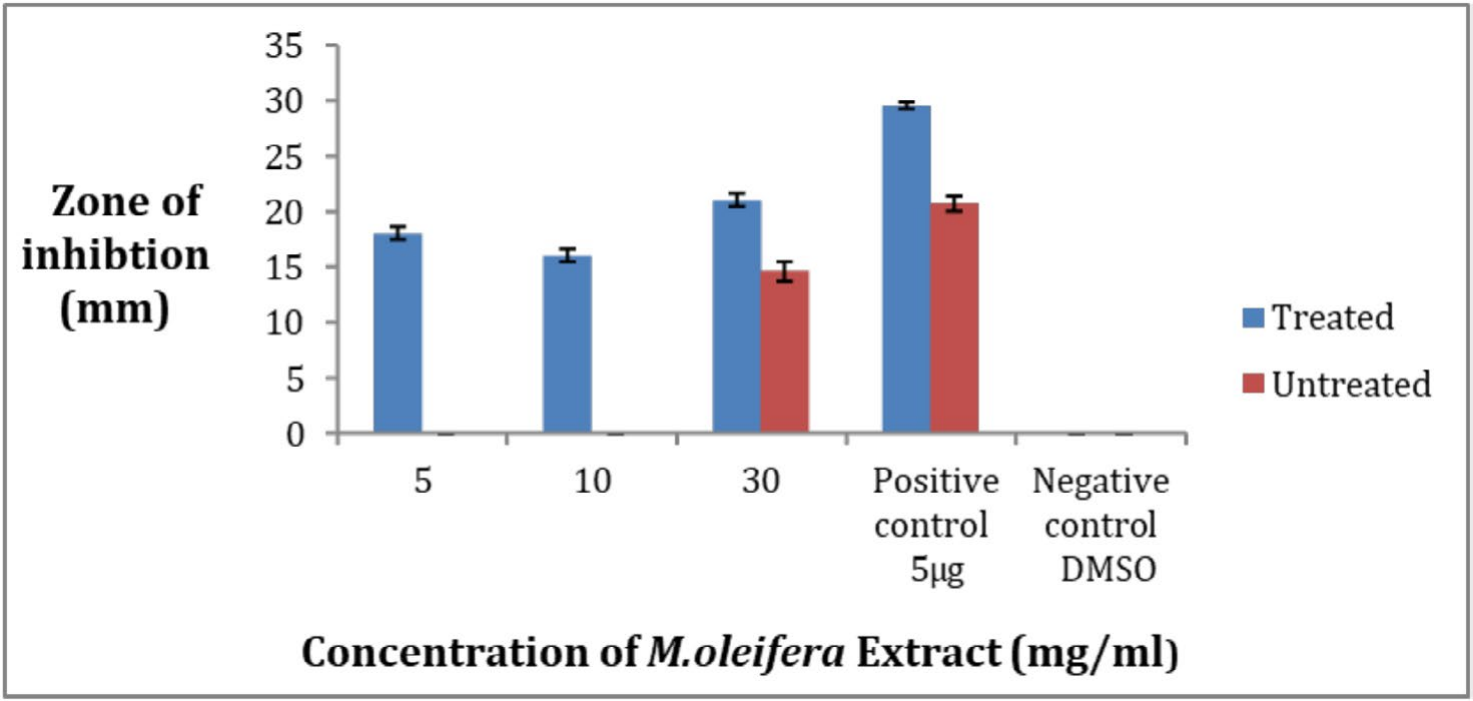
Zone of inhibition of *S. aureus* strain by crude extract of *Moringa oleifera* .

Against pathogenic *S. aureus* strain the crude extract of both ozone‐treated and untreated leaves of *M. oleifera* showed activity at concentrations of 5, 10 and 30 mg/mL in comparison to the antibiotic ciprofloxacin as shown in Figure 15. In ozone‐stressed *M. oleifera* leaves the maximum zone of inhibition was 21 ± 1 mm at 30 mg/mL while the minimum zone of inhibition was 16 ± 1 mm at 10 mg/mL. In untreated leaves extract the maximum zone was 14.6 ± 1.5 mm while there was no zone of inhibition at the other two concentrations (Figure 16). All the values were taken in replicates. The negative control did not give any value.

### 3.13 Comparison of Antibacterial Activity of Ozone‐Stressed and Untreated *M. oleifera* 's Crude Extract for *P. aeruginosa* at the Concentrations 5, 10, and 30 mg/mL

**FIGURE 17:**
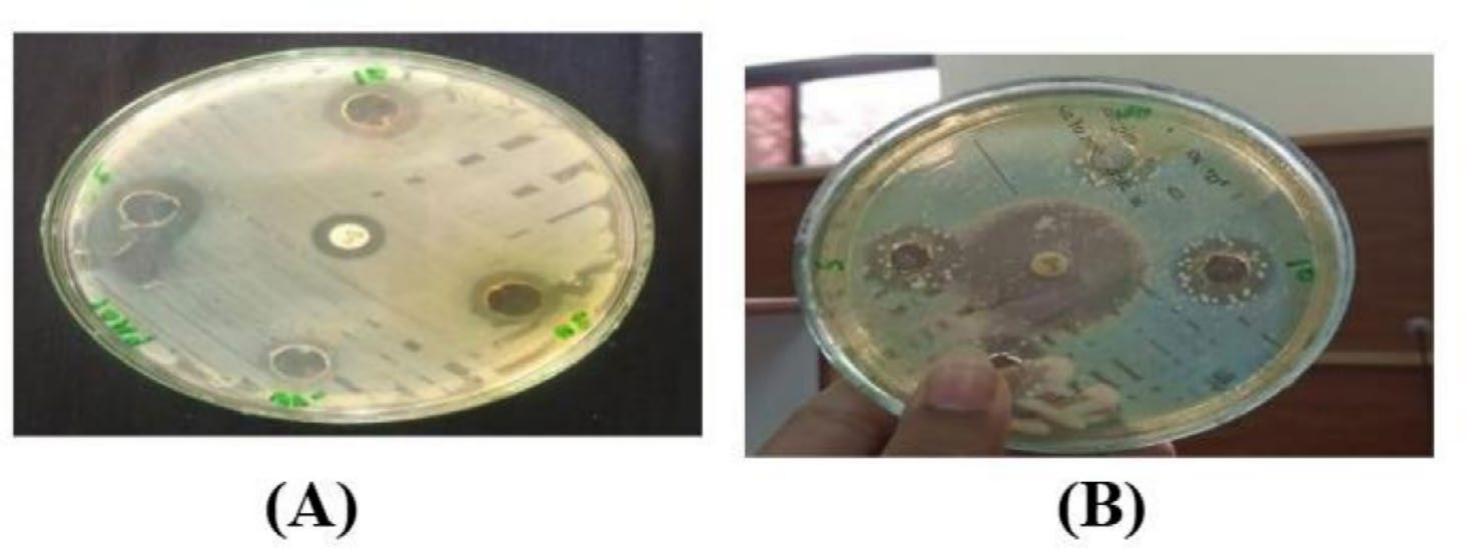
Zone of inhibition of *P. aeruginosa* strain by crude extract of *Moringa oleifera* at concentrations of 5, 10, and 30 mg/mL.

**FIGURE 18:**
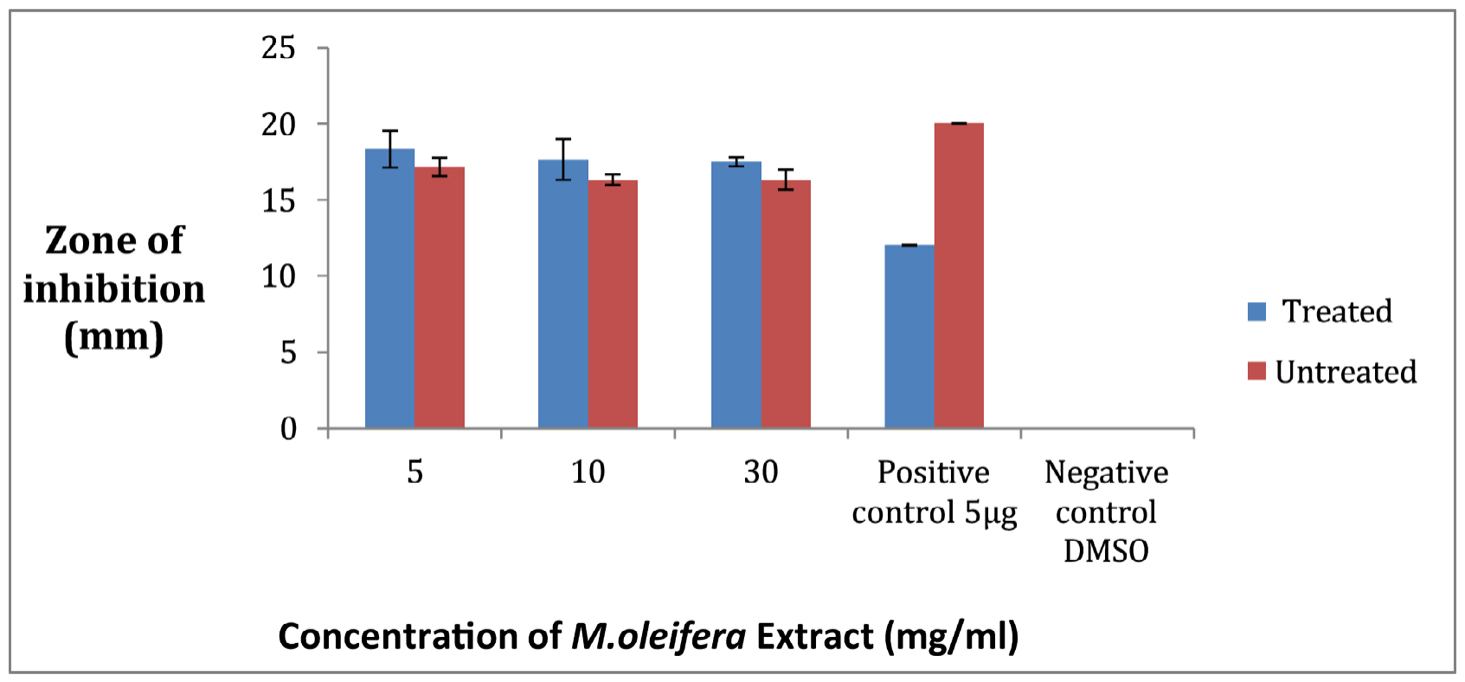
Zone of inhibition of *P. aeruginosa* strain by crude extract of *Moringa oleifera* .

Against pathogenic *P. aeruginosa* strain the crude extract of both ozone‐treated and untreated leaves of *M. oleifera* showed activity at concentrations of 5, 10 and 30 mg/mL in comparison to the antibiotic ciprofloxacin (Figure 17). In ozone‐stressed *M. oleifera* leaves the maximum zone of inhibition was 18.33 ± 2 mm at 5 mg/mL while the minimum zone of inhibition was 17.5 ± 0.5 mm at 30 mg/mL. In untreated leaves extract the maximum zone was 17.1 ± 1 mm at 5 mg/mL while both other concentrations gave the same values of 16.3 ± 0.5 mm as shown in Figure 18. All the values were taken in replicates. The negative control did not give any value.

### 3.14 Comparison of Antibacterial Activity of Ozone‐Stressed and Untreated *M. oleifera* 's Crude Extract for *S. aureus* at the Concentrations 50, 100, and 300 mg/mL

**FIGURE 19:**
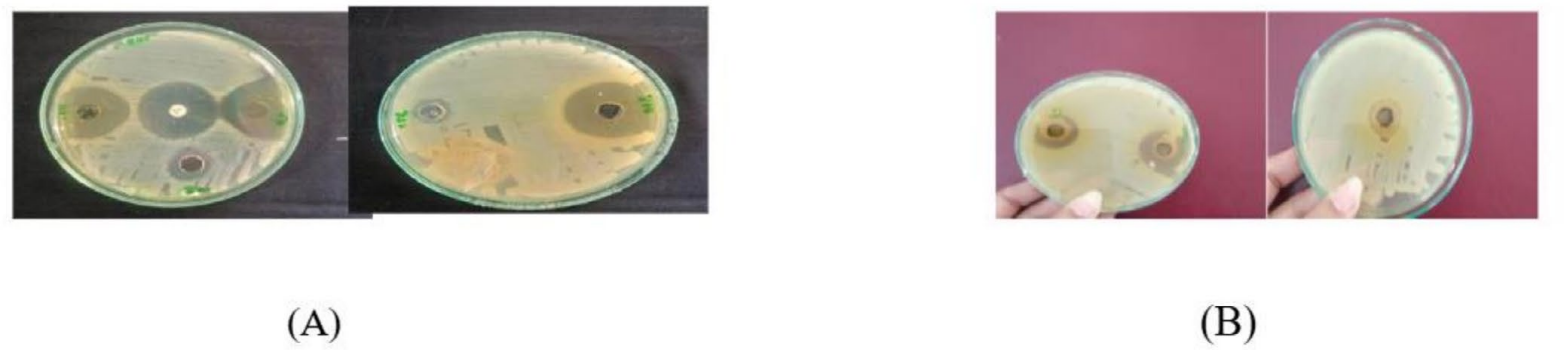
Zone of inhibition at 50, 100, and 300 mg/mL of *M. oleifera* crude extract for pathogenic strain, *S. aureus* (A: treated, B: untreated).

**FIGURE 20:**
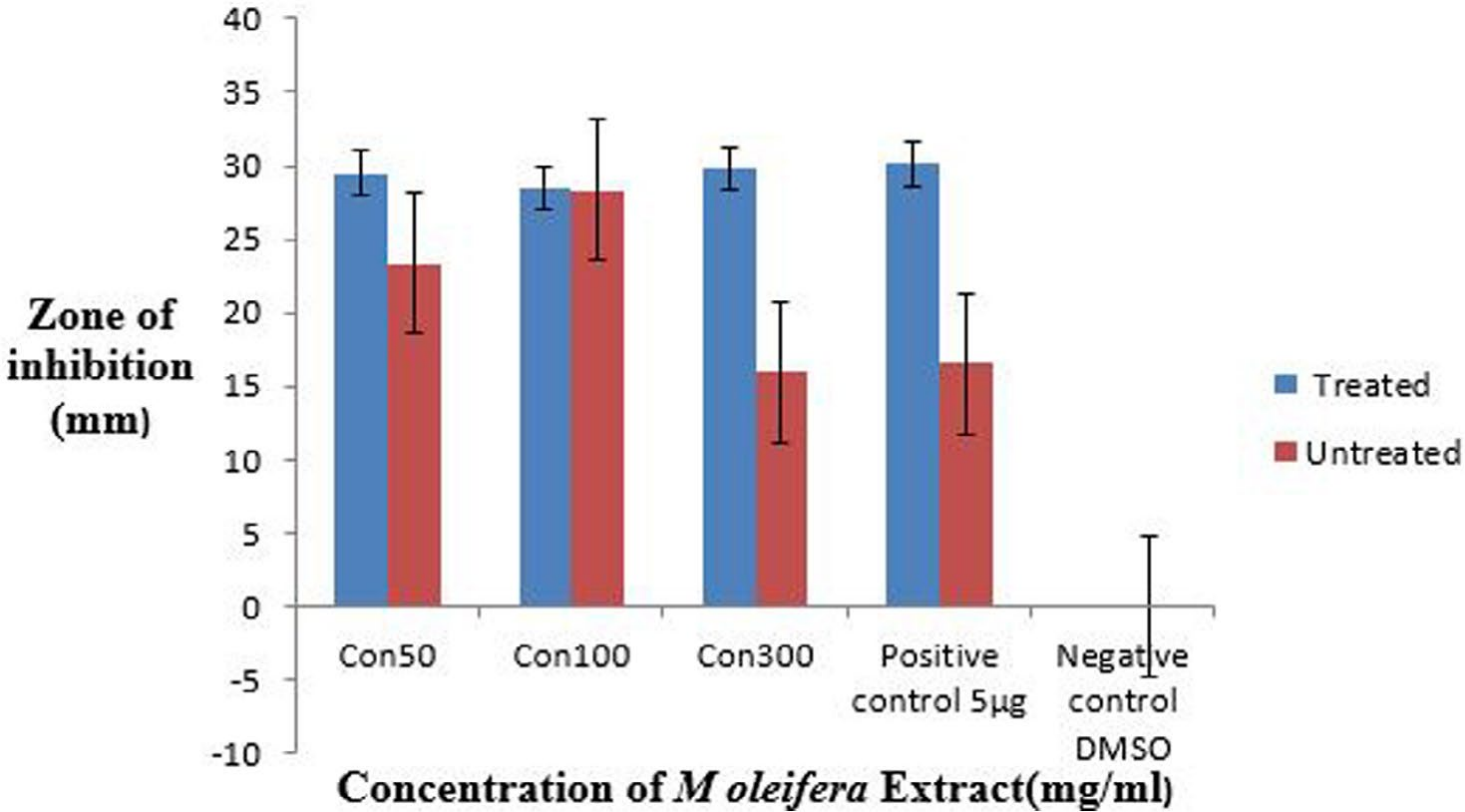
Zone of inhibition of pathogenic strain by crude extract of *Moringa oleifera* .

The crude methanolic extract of ozone‐treated and untreated leaves of *M. oleifera* showed activity against *S. aureus* as shown in Figure 19. For both treatments the concentrations were 50,100 and 300 mg/mL taken in comparison to the antibiotic ciprofloxacin. The maximum zone of inhibition was 29.833 ± 0.2 mm at 300 mg/mL while the minimum zone was 28.5 ± 0.5 mm at 100 mg/mL in the case of treated leaves extract while the maximum zone was 28 ± 1 mm at 100 mg/mL, and the minimum zone was 16 ± 1 mm at 300 mg/mL in the case of untreated leaves extract (Figure 20). All the values were taken in triplicates. The negative control did not give any value.

### 3.15 Comparison of Antibacterial Activity of Ozone‐Stressed and Untreated *M. oleifera* 's Crude Extract for *P. aeruginosa* at the Concentrations 50, 100, and 300 mg/mL

**FIGURE 21:**
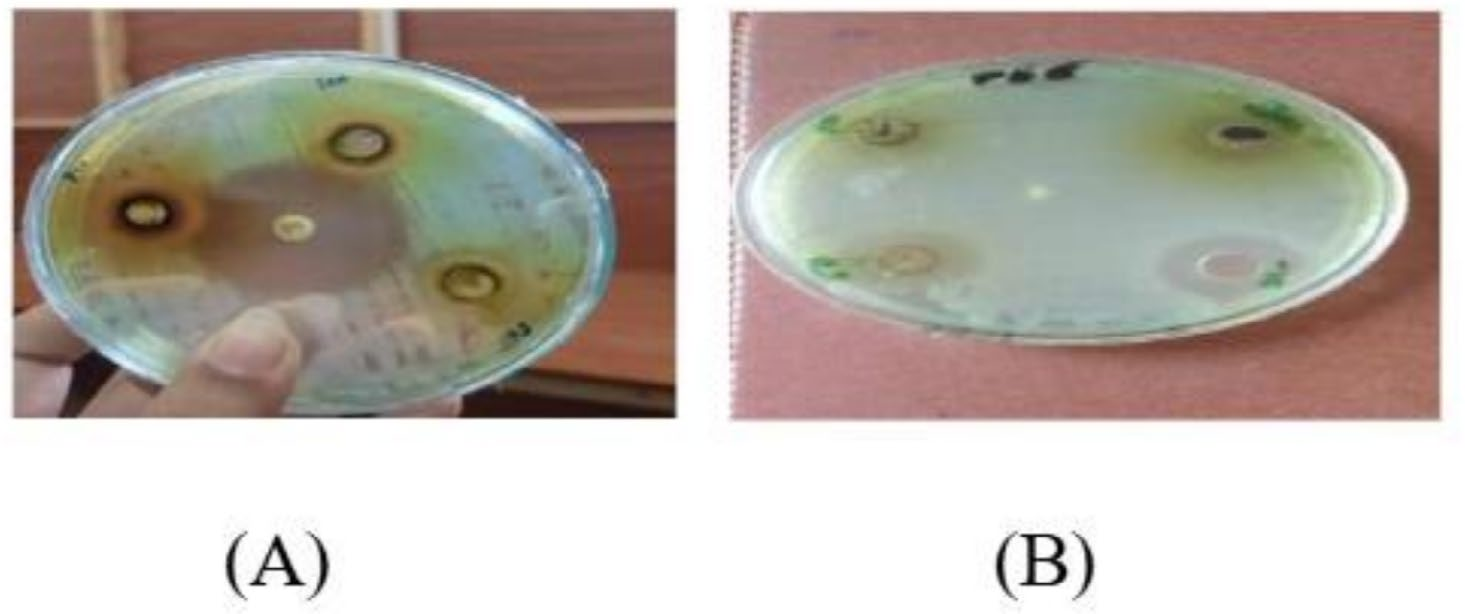
Zone of inhibition at 50, 100, and 300 mg/mL of *M. oleifera* crude extract.

**FIGURE 22:**
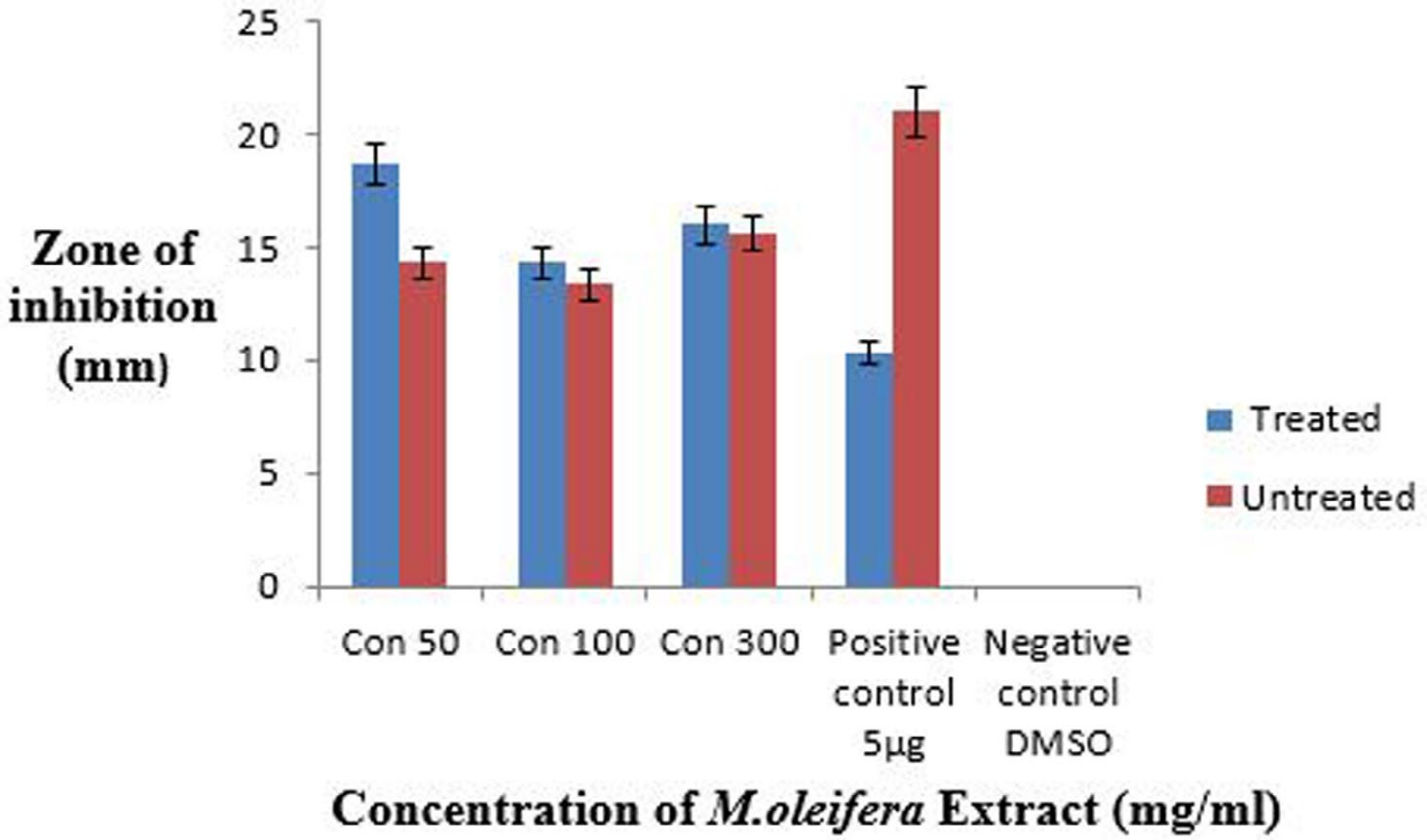
Zone of inhibition of pathogenic strain by crude extract of *Moringa oleifera* .

The crude methanolic extract of ozone‐treated and untreated leaves of *M. oleifera* showed activity against *P. aeruginosa* . For both treatments the concentrations were 50, 100, and 300 mg/mL taken in comparison to the antibiotic ciprofloxacin (Figure 21). The maximum zone of inhibition was 18.6 ± 1.5 mm at 50 mg/mL while the minimum zone was 14.3 ± 2 mm at 100 mg/mL in the case of the treated leaves extract whereas the maximum zone was 15.6 ± 1.1 mm at 100 mg/mL, and the minimum zone was 13.3 ± 1.1 mm at 300 mg/mL in the case of the untreated leaves extract as shown in Figure 22. All the values were taken in triplicates. The negative control did not give any value.

### 3.16 Phytochemical Analysis

The selected medicinal plants were screened for their polyphenol contents and the results are given in the Table 1.

**TABLE 1:** Total phenolics and flavonoids contents in selected medicinal plant.

Scientific name	Plant code	TPC (mgGAE/100 g, FW)	TFC (mgQAE/100 g, FW)
*Moringa oleifera* (ozone stressed plant)	OP	69.76 ± 0.4	236.0 ± 0.4
*Moringa oleifera* (untreated plant)	UP	64.29 ± 0.3	180 ± 2.7

*Note:* Values are the means of triplicates results ± SD.

Abbreviations: GAE, gallic acid equivalent; QDE, quercetin dihydrate equivalent; TFC, total flavonoids content; TPC, total phenolics content.

#### 3.16.1 Total Phenolic and Flavonoid Contents

Total phenolic contents of ozone‐stressed and untreated plants were measured in this study. The highest amounts of TPC were found in ozone‐stressed plants as compared to normal untreated plants. Different concentrations of polyphenols were reported and significant differences were found between selected plants. Total phenolic and flavonoid contents of ozone‐stressed *M. oleifera* and untreated *M. oleifera* plants were measured in this study. In comparison of both treated and untreated plants, the level of TPC in treated plants was higher than that in untreated plants, OP > UP. The highest amount of TFC was also observed in the same manner OP > UP (Table 1).

To analyze our experimental data—including zone of inhibition, total phenolic content (TPC), and total flavonoid content (TFC)—we used one‐way ANOVA. To compare differences between groups, we followed up with Tukey's test and the Least Significant Difference (LSD) test, using a significance level of *p* = 0.05.

### 3.17 Docking Results

Molecular docking results help us understand how a compound—like a secondary metabolite—might interact with a specific protein, giving clues about how well and how specifically they bind. The docking table usually lists values such as *S* (kcal/mol), which shows the binding energy; the more negative this number, the stronger the predicted interaction. RMSD (Å) tells us how closely the predicted binding pose matches a known or ideal structure—lower values (typically below 2 Å) suggest a better and more reliable fit. It also lists the interacting residues, which are the amino acids in the protein that come into contact with the compound. The images that go along with the docking results help visualize these interactions, showing whether the compound is binding with acidic, basic, polar, or non‐polar residues. Each type of residue points to different kinds of interactions—like hydrogen bonds, ionic bonds, or hydrophobic effects. Together, these results illustrate the binding mode, stability, and specificity of the ligand–protein complex, offering a foundation for drug design or understanding molecular mechanisms (Meng et al. 2011; Morris et al. 2009).

For the molecular docking part of the study, we used a combination of tools: AlphaFold to generate the 3D structures of the proteins, PubChem to obtain structures of FDA‐approved phenolic and flavonoid compounds, and MOE (Molecular Operating Environment) to carry out the docking and calculate docking scores.

#### 3.17.1 Staphylokinase (SAK)

**FIGURE 23:**
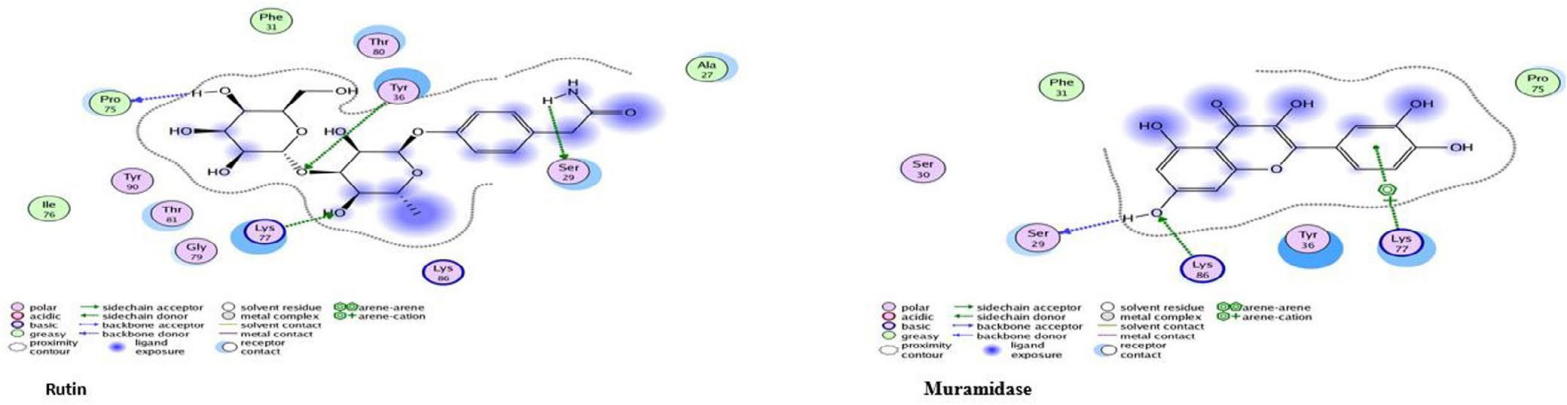
Two‐dimensional interactions of *S. aureus* staphylokinase protein‐ ligand docked complexes with least *S* values (kcal/mol).

Staphylokinase (SAK) is one of the *S. aureus* virulence factors that damage tissue and increase bacterial invasiveness (Marchica et al. 2021). Staphylokinase docked with 30 secondary metabolite compounds of *Moringa*; among the tested compounds, Rutin exhibited the strongest binding energy (−15.56 kcal/mol), Overlapping residues such as Ser29, Tyr36, and Lys77 indicate key binding hotspots on the SAK protein. Among the metabolites, Rutin and Marumoside B show strong potential as anti‐staphylococcal agents. The best docking complexes are illustrated in Figure 23 whereas docking findings with the active site residues are reported in Table 2.

**TABLE 2:** Docking analyses of *Staphylococcus aureus* staphylokinase protein with secondary metabolites.

S. No.	Tested secondary metabolites	*S* (kcal/mol)	RMSD_refine	Interacting residues
3	Rutin	−15.5559	3.037321	Ser29, Lys77, Lys86
9	Marumoside B (66)	−13.1467	1.444988	Ser29, Tyr36, Pro75, Lys77
32	Lutein Xanthein	−13.6547	1.967541	Tyr36, Lys86

*Note:* *S* score of placement of polyphenol inside binding pocket of the protein using London dG scoring function. RMSD_refine, root‐mean‐square deviation between atoms of predicted pose (after refinement) and those of crystal structure (before refinement); n.d. not detected; Ignored: MOE could not draw the protein‐ligand complex.

#### 3.17.2 Clumping Factor

**FIGURE 24:**
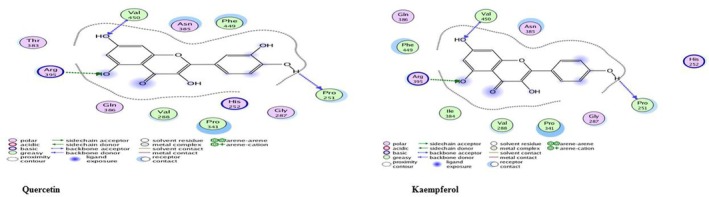
Two‐dimensional interactions of *S. aureus* clumping factor A protein‐ligand docked complexes with least *S* values (kcal/mol).

*S. aureus* A (ClfA), a cell‐wall‐anchored protein, is a virulence factor in many infections and promotes the colonization of protein‐coated biomaterials (Herman‐Bausier et al. 2018). Myricetin showing the highest binding energy (−19.10 kcal/mol), Arg395 consistently appeared as a key interacting residue across most compounds, suggesting it may serve as a crucial binding hotspot in Clf‐mediated host‐pathogen interactions. The low RMSD values (0.58–1.55 Å) across all compounds confirm the stability and reliability of these binding interactions. These findings support the potential of *M. oleifera* flavonoids—particularly Myricetin, Luteolin, and Rutin—as promising inhibitors targeting the clumping factor of *S. aureus*; docking findings with active site residues are reported in Table 3, and the best docking complexes are displayed in Figure 24.

**TABLE 3:** Docking analyses of *Staphylococcus aureus* clumping factor A protein with secondary metabolites.

S. No.	Tested secondary metabolites	*S* (kcal/mol)	RMSD_refine (Å)	Interacting residues
1	Quercetin	−17.4535	0.589256	Pro251, Arg395, Val450
2	Kaempferol	−17.218	0.877656	Pro251, Arg395, Val450
3	Rutin	−18.3043	1.552224	Pro251, Ile384, Asn525
4	Isorhamnetin	−17.7377	1.307003	Arg395, Val 450
5	Luteolin	−18.3983	0.742895	Arg395, Ser447
7	Myricetin	−19.1031	1.25805	Arg395

*Note:* *S* score of placement of polyphenol inside binding pocket of the protein using London dG scoring function. RMSD_refine, root‐mean‐square deviation between atoms of predicted pose (after refinement) and those of crystal structure (before refinement); n.d. not detected; ignored: MOE could not draw the protein‐ligand complex.

### 3.18 Alkaline Protease

**FIGURE 25:**
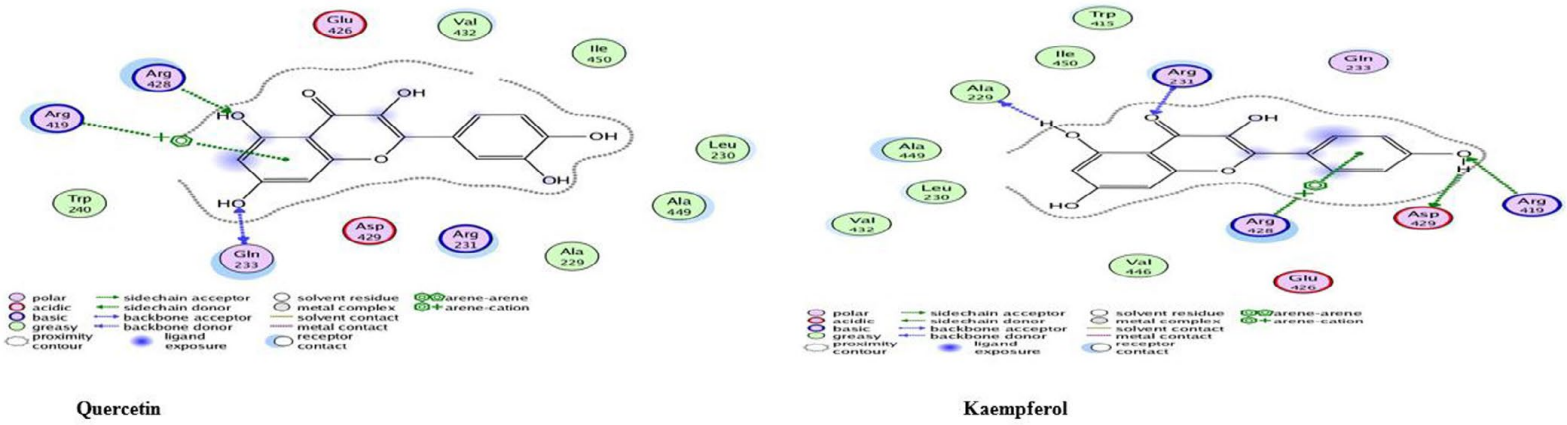
Two‐dimensional interactions of *P. aeruginosa* alkaline protease protein‐ligand docked complexes with least *S* values (kcal/mol).

*P. aeruginosa* 's alkaline protease (AprA) is known to prevent complement‐mediated lysis of erythrocytes, (Laarman et al. 2012). Rutin showed the highest binding energy (−17.84 kcal/mol), suggesting strong interaction and inhibitory potential A key interacting residue, Arg419, was consistently involved across all ligands, indicating it may serve as a critical binding hotspot. These findings suggest that *M. oleifera* compounds, particularly Rutin, may effectively inhibit the alkaline protease of *P. aeruginosa* , offering potential for anti‐virulence therapeutic development, the Table 4 lists the docking outcomes using the active site residues. Although the best docking complexes are depicted in Figure 25.

**TABLE 4:** Docking analysis of *Pseudomonas aeruginosa* alkaline protease with secondary metabolites.

S. No.	Tested secondary metabolites	*S* (kcal/mol)	RMSD_refine (Å)	Interacting residues
1	Quercetin	−14.7211	1.158413	Arg419, Arg428, Gln 233
2	Kaempferol	−14.6745	1.595999	Ala229, Arg231, Arg419
3	Rutin	−17.8401	1.869492	Arg231, Gln233, Arg419

*Note:* *S* score of placement of polyphenol inside the binding pocket of the protein using London dG scoring function. RMSD_refine, root‐mean‐square deviation between atoms of predicted pose (after refinement) and those of crystal structure (before refinement); n.d. not detected; Ignored: MOE could not draw the protein‐ligand complex.

#### 3.18.1 Elastase B Protein

**FIGURE 26:**
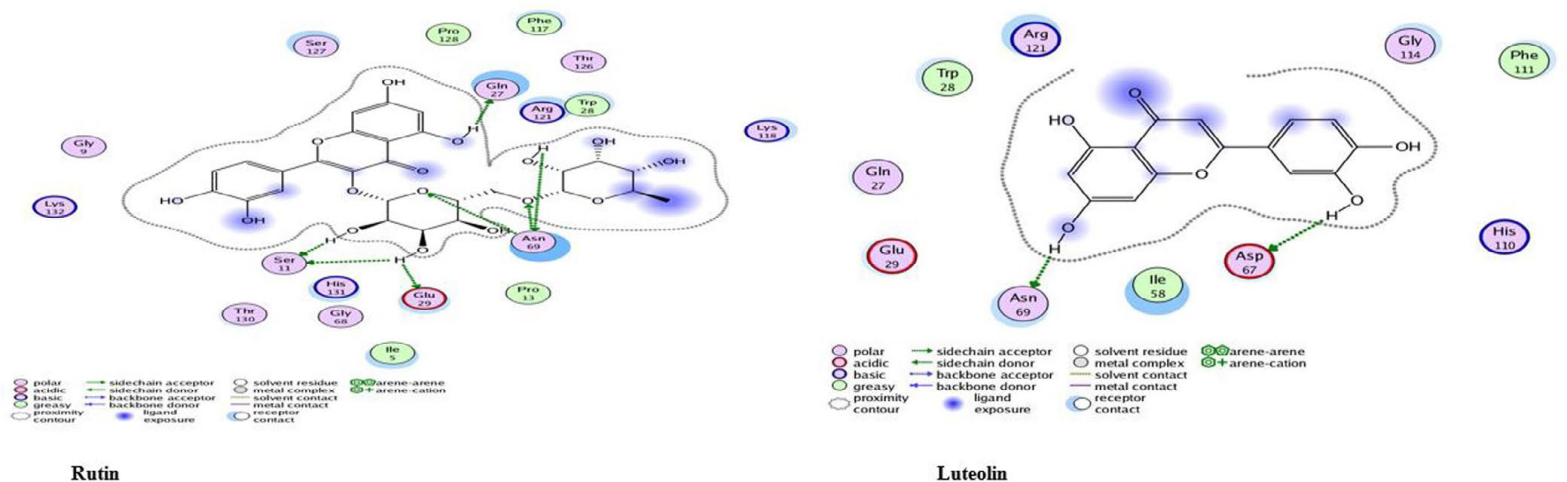
Two‐dimensional interactions of *P. aeruginosa* Elastase B protein‐ligand docked complexes with least *S* values (kcal/mol).

Elastase B is a virulent protein docked with 30 secondary metabolites of *Moringa*, Rutin exhibiting the highest binding affinity (−17.30 kcal/mol). Residue Asn69 was a common interaction site across all tested compounds, indicating a potential binding hotspot crucial for inhibitor binding. These results suggest that *M. oleifera* flavonoids, particularly Rutin, hold promise as potential inhibitors of elastase B, a key virulence factor of *P. aeruginosa* , Docking results with the active site residues are given in Table 5. Whereas, best docking complexes are shown in Figure 26.

**TABLE 5:** Docking analyses of *Pseudomonas aeruginosa* elastase B protein with secondary metabolites.

S. No.	Tested secondary metabolites	*S* (kcal/mol)	RMSD_refine (Å)	Interacting residues
3	Rutin	−17.3019	1.809411	Ser11, Gln27, Glu29, Asn69
5	Luteolin	−16.7456	1.738716	Asp67, Asn69
7	Myricetin	−16.544	1.860589	Asp67, Asn69
9	Marumoside B (66)	−13.4842	3.017948	Asn69, His131

*Note:* *S* score of placement of polyphenol inside binding pocket of the protein using London dG scoring function. RMSD_refine, root‐mean‐square deviation between atoms of predicted pose (after refinement) and those of crystal structure (before refinement); n.d. not detected; Ignored: MOE could not draw the protein‐ligand complex.

## 4 Discussion

Different bacterial strains can cause a wide variety of illnesses, many of which are fatal to humans. As a hospital pathogen, *P. aeruginosa* causes infectious wounds, burns, and infectious diseases like meningitis, urinary tract infections, and so forth (Mena and Gerba 2009). *S. aureus* is a major pathogen that is prevalent everywhere, especially in pediatric wards, intensive care units, operating rooms, and chemotherapy clinics. It can cause a variety of infections, including food poisoning and life‐threatening infections, when it directly infects wounds, like *Staphylococcus* wound infection after surgery (Ondusko and Nolt 2018).

For the treatment of such infections antibiotics had been used since 1928, but due to the repetitive use of antibiotics such infectious pathogens became resistant and commonly used antibiotics are useless. One of the biggest issues in treating and controlling the infection is antibiotic resistance. The usage of antimicrobials with this resistance pattern has been viewed as a serious concern in the medical community (Ondusko and Nolt 2018). Nature has developed many solutions or remedies to cure the diseases caused by bacteria. One of these natural remedies is the antimicrobial activity of plants. Plants produce different secondary bioactive compounds that are well known for their therapeutic properties which have no side effects compared to synthetic alternatives.

Over the last 10 years, researchers have attempted to produce nanoparticles from various plant components, as well as the forms and sizes of nanoparticles have been changed through engineering to achieve increased activity of nanoparticles in various fields like biomedical, antibacterial, and so forth. Therefore, in the current work, we emphasized the evaluation of the therapeutic potential of medicinal plants (normal and ozone stressed) and their bioactive AgNPs. *Moringa oleifera* was chosen as this plant has ethnomedicinal and traditional uses, which indicate that the plant has pleiotropic therapeutic efficacy against human pathogens (Ghimire et al. 2024). The broad therapeutic potential of plant‐based treatments is well documented, and our study contributes to this growing body of evidence by showing enhanced efficacy of ozone‐stressed *Moringa* extracts combined with AgNPs (Luo et al. 2024). Fresh leaves were dissolved in methanol to obtain plant extract for the purpose of AgNPs synthesis. The extract was added to Ag solution; after 24 h the color of the mixture turned brownish from green, which indicated the production of Ag nanoparticles. A full change in color showed the reaction was complete.

The carbonyl and hydroxyl groups of flavonoids have roles in the biosynthesis of AgNPs and serve a significant part in the lowering of silver ions by chelating metal ions with the flavonoids, where the charge transport and electrostatic relationship between the OH group of flavonoids and silver ions is crucial for the metabolic action of the process of reduction. Flavan‐3‐ol, Flavan‐4‐ol, and Flavon‐3, 4‐diol have been demonstrated to be efficient as lowering or stabilizing agents in reducing the amount of Ag+ to Ag0 (Habeeb Rahuman et al. 2022). It has been reported that flavonoids showed up to six fold more antibacterial activities than standard drugs. In addition, some synthetic derivatives of flavonoids also exhibited remarkable antibacterial activities with 20 to 80‐fold more potent activity than the standard drug against multidrug‐resistant gram‐negative and gram‐positive bacteria (Farhadi et al. 2019) AgNPs interact with the viral surface protein in enveloped and nonenveloped viruses. In addition, AgNPs block the penetration of viruses into the host cell as well as AgNPs block the cellular viral entry to the nucleus and inhibit viral replication by blocking the viral genome. AgNPs synthesized utilizing *Phyllanthus emblica* leaf extract showed anticancer activity against hepatocellular carcinoma (Jain et al. 2021). The size and surface charge of NPs can be identified by zeta potential. As revealed from the graph, ozone‐stressed nanoparticles have a charge of −23 mV while the size is 131 ± 9 nm. The charge of normal plant nanoparticles is −30 mV while the size is found to be 241 ± 2.04 nm. The small size and negative charge nanoparticles show stability. The aqueous extract of *Moringa* leaves (normal and ozone stressed) showed antibacterial activity against the selected pathogens, ( *P. aeruginosa* and *S. aureus* ) (Zhang et al. 2021). The agar well diffusion method was used to check the antibacterial activity of *P. aeruginosa* and *S. aureus* . Both strains were inhibited by the aqueous extract of leaves (normal and ozone stressed). *Moringa* leaves extract showed antibacterial activity as well as reported by (Fouad et al. 2019). Bacteria cause many contagious diseases and for the cure of those diseases antibiotics have been used for decades, but due to the repetitive use of antibiotics the pathogenic bacteria developed resistance against these antibiotics and made them less effective against these pathogenic strains. Due to the effectiveness of plant‐ based NPs as antibiotics, biological studies on the antimicrobial role of nanoparticles have increased. Medicinal plants also possess antibacterial activity but the major problem is bioavailability.

Therefore, in this research medicinal plant‐based AgNPs' antibacterial potential was investigated against gram‐positive and gram‐negative bacteria by agar well diffusion method. Ag nanoparticles consist of small size, high specific surface area (the ratio of free surface area to mass), high reactivity and bioavailability. The ultra‐small size of AgNPs and, as a consequence, a high specific surface area is one of the main reasons for their increased antibacterial activity (Verkhovskii et al. 2019; Liu et al. 2025). Leaves‐based AgNPs from *Moringa* showed antibacterial activity against *P. aeruginosa* and *S. aureus* . The antibacterial potency of ozone‐stressed plant nanoparticles was higher than that of normal plant nanoparticles. The ozone‐stressed plants‐based AgNPs inhibited the growth of *P. aeruginosa* and *S. aureus* even at lower concentrations. In the case of *P. aeruginosa* AgNPs gave zones of inhibition but zones were bigger in ozone‐stressed leaves‐based AgNPs than in normal leaves‐based AgNPs at all concentrations. The ozone‐stressed leaves‐based AgNPs have more potential for antibacterial activity than normal leaves‐based AgNPs; this could be due to the presence of a higher concentration of plant antioxidants (e.g., secondary metabolites and other bioactive compounds), which are normally produced to cope with O_3_‐induced oxidative stress (Marchica et al. 2021).

According to the results, ozone‐stressed leaves‐based AgNPs inhibited the growth of *S. aureus* while normal leaves‐based AgNPs did not give any zone of inhibition. In comparison of both strains the zones of inhibition were bigger in *P. aeruginosa* than *S. aureus* In general gram‐negative bacteria were more sensitive to AgNPs than gram‐positive bacteria. Silver nanoparticles (AgNPs) are more effective against gram‐negative bacteria. Gram‐negative bacteria have smaller cellular walls than gram‐positive ones. The strong cellular membrane could prevent nanoparticles from entering cells (Marchica et al. 2021).

## 5 Conclusions

Ozone treated and normal *M. oleifera* aqueous extract had been used to synthesize the Ag nanoparticles in an environmentally friendly manner. *M. oleifera* consists of many important secondary metabolites which help to reduce and stabilize the nanoparticles; therefore the potentially hazardous substances were minimized. Ag nanoparticles were characterized in order to determine the optical and morphological properties of the nanoparticles. Human pathogenic strains *S. aureus* and *P. aeruginosa* were used to investigate the antibacterial activity of NPs and the crude extract of the plants.

Ozone‐treated plants gave better results in both strains than normal plants. The crude extract of ozone‐treated plants showed maximum activity against both strains as compared to normal plant crude extract. Ozone‐treated plant NPs gave antibacterial activity against both strains while untreated/normal plant NPs inhibited only *P. auruginosa*, and they did not show any activity against *S. aureus*. Therefore, AgNPs are effective therapeutic agents but the ozone‐treated NPs are even more effective than normal plant AgNPs that can be used for a variety of biomedical applications, which is a promising field that may be investigated in the future. For in vivo research AgNPs can be used as NPs have higher antibacterial activity than crude extract. Further, in silico docking of virulent proteins with the phytocompounds produced stable complexes, These findings suggest that the compounds have strong antimicrobial potential against harmful pathogens. Further in vivo studies on selected plants could pave the way for developing more targeted and effective antimicrobial drugs in the future.

## Author Contributions

**Misbah Zaid Ali:** conceptualization (equal), investigation (equal), methodology (equal), writing – original draft (equal). **Zia Ullah:** data curation (equal), methodology (equal). **Sabaz Ali Khan:** formal analysis (equal), visualization (equal). **Rafiq Ahmad:** data curation (equal), investigation (equal). **Nadia Bibi:** methodology (equal), validation (equal). **Hira Mushtaq:** software (equal), validation (equal). **Bushra Rehman:** resources (equal), visualization (equal). **Qamar Sajjad:** project administration (equal), supervision (equal). **Awais Raza:** data curation (equal), formal analysis (equal). **Agoura Diantom:** investigation (equal), writing – review and editing (equal).

## Consent

All authors agree to publish.

## Conflicts of Interest

The authors declare no conflicts of interest.

## Data Availability

The data that support the findings of this study are available from the corresponding author upon reasonable request.
